# Efficient activation of the lymphangiogenic growth factor VEGF-C requires the C-terminal domain of VEGF-C and the N-terminal domain of CCBE1

**DOI:** 10.1038/s41598-017-04982-1

**Published:** 2017-07-07

**Authors:** Sawan Kumar Jha, Khushbu Rauniyar, Terhi Karpanen, Veli-Matti Leppänen, Pascal Brouillard, Miikka Vikkula, Kari Alitalo, Michael Jeltsch

**Affiliations:** 1 Translational Cancer Biology Research Program, Biomedicum Helsinki, University of Helsinki, PO Box 63, Haartmaninkatu 8, Helsinki, 00014 Finland; 2 Wihuri Research Institute, Biomedicum Helsinki, University of Helsinki, Helsinki, Finland; 3 Helsinki University Central Hospital, Helsinki, Finland; 4 University Hospital Radiumhospitalet and K. G. Jebsen Center for Cancer Immunotherapy, Institute for Clinical Medicine, University of Oslo, Oslo, Norway; 5 de Duve Institute, University of Louvain, Brussels, Belgium; 6 Walloon Excellence in Life Sciences and Biotechnology (WELBIO), University of Louvain, Brussels, Belgium

## Abstract

The collagen- and calcium-binding EGF domains 1 (CCBE1) protein is necessary for lymphangiogenesis. Its C-terminal collagen-like domain was shown to be required for the activation of the major lymphangiogenic growth factor VEGF-C (Vascular Endothelial Growth Factor-C) along with the ADAMTS3 (A Disintegrin And Metalloproteinase with Thrombospondin Motifs-3) protease. However, it remained unclear how the N-terminal domain of CCBE1 contributed to lymphangiogenic signaling. Here, we show that efficient activation of VEGF-C requires its C-terminal domain both *in vitro* and in a transgenic mouse model. The N-terminal EGF-like domain of CCBE1 increased VEGFR-3 signaling by colocalizing pro-VEGF-C with its activating protease to the lymphatic endothelial cell surface. When the ADAMTS3 amounts were limited, proteolytic activation of pro-VEGF-C was supported by the N-terminal domain of CCBE1, but not by its C-terminal domain. A single amino acid substitution in ADAMTS3, identified from a lymphedema patient, was associated with abnormal CCBE1 localization. These results show that CCBE1 promotes VEGFR-3 signaling and lymphangiogenesis by different mechanisms, which are mediated independently by the two domains of CCBE1: by enhancing the cleavage activity of ADAMTS3 and by facilitating the colocalization of VEGF-C and ADAMTS3. These new insights should be valuable in developing new strategies to therapeutically target VEGF-C/VEGFR-3-induced lymphangiogenesis.

## Introduction

Vascular endothelial growth factor C (VEGF-C), the major effector of lymphangiogenesis, is indispensable for lymphatic development in mouse embryos and essential for most lymphangiogenic processes in adults^1–3^. VEGF-C mediates its signals by binding to and activating the vascular endothelial growth factor receptors VEGFR-3 and VEGFR-2^4^. VEGF-C is synthesized as a precursor molecule, in which the central VEGF homology domain (VHD) is flanked by amino (N)-terminal and carboxy (C)-terminal propeptides. Both propeptides are proteolytically removed during the generation of the active (“mature”) form of VEGF-C^4^. The affinity of VEGF-C towards the lymphangiogenic receptor VEGFR-3 increases with each proteolytic cleavage and the resulting mature protein can also activate the major angiogenic receptor VEGFR-2^4^.

Studies on a VEGF-C mutant lacking the C-terminal propeptide (*vegfc* ^um18^) suggest a role of the C-terminal propeptide in lymphatic development^5^, and a similar mutation was found in a patient with Milroy-like lymphedema^6^. However, as the VEGF-C^um18^ mutant also displays a secretion defect, the associated phenotype may not reflect all functions of the C-terminal propeptide. Adenoviral delivery of a chimeric protein, in which the N- and C-terminal propeptides of VEGF-C flanked the VHD of VEGF-A, resulted in a denser and more fine-meshed lymphatic capillary network than VEGF-A_165_, although the detailed mechanism has not been reported^7^.

Mutations that perturb lymphangiogenic signaling have been found in VEGFR-3 and its ligand VEGF-C; these mutants cause hereditary lymphedema type 1 A and 1D, respectively^8, 9^. Recently, mutations in the *CCBE1* (collagen- and calcium-binding epidermal growth factor domains 1) gene were found in a subset of patients with Hennekam lymphangiectasia-lymphedema syndrome^10^. Consistent with these data, deletion of either *Ccbe1* or *Vegfc* in mice completely halts lymphatic vasculature development at embryonic day (E) 10.5^11–13^.

CCBE1 is a secreted protein containing an N-terminal domain with three calcium binding EGF-like repeats and a C-terminal domain with two collagen-like repeats^10^. Most known mutations of CCBE1 in human patients localize to its N-terminal EGF-like repeats^10, 14^. In zebrafish and cell culture models, mutations in the N-terminal domain are better tolerated than mutations in the C-terminal domain^10, 15^. Recent studies revealed that a disintegrin and metalloproteinase with thrombospondin motifs-3 (ADAMTS3) activates VEGF-C most efficiently in a complex with CCBE1^11, 15, 16^. The phenotype of the *Adamts3*-deleted mice has further confirmed the importance of ADAMTS3 for lymphatic development^17^. ADAMTS3-mediated activation of VEGF-C is accelerated by the C-terminal collagen-like domain of CCBE1^15^. Both *Ccbe1* null mice and mice devoid of the Ccbe1 C-terminal collagen-like domain showed a complete lack of lymphatic structures, whereas mice devoid of the Ccbe1 N-terminal EGF-like domain showed some clusters of lymphatic endothelial cells (LECs), which were unable to form contiguous structures, suggesting that the CCBE1 N-terminal domain is involved in the organization and migration of LECs^15^.

We have previously shown that the CCBE1-mediated activation of pro-VEGF-C can occur directly on the surface of endothelial cells^16^. As pro-VEGF-C interacts with heparan sulfate proteoglycans (HSPGs)^18^ we speculated that the colocalization of pro-VEGF-C, CCBE1, and ADAMTS3 on the cell surface may be necessary for efficient cleavage of pro-VEGF-C to generate mature, active VEGF-C and that the N-terminal domain of CCBE1 and the C-terminal domain of VEGF-C could play a central role in this colocalization.

In this study, we analyzed the distribution of VEGF-C, CCBE1, and ADAMTS3 after secretion, the involvement of different domains in the localization of these proteins, and the effects of domain deletion mutants of VEGF-C and CCBE1 on VEGFR-3 activation.

## Results

### VEGF-C binds to extracellular matrix via its C-terminal domain

**Figure 1:**
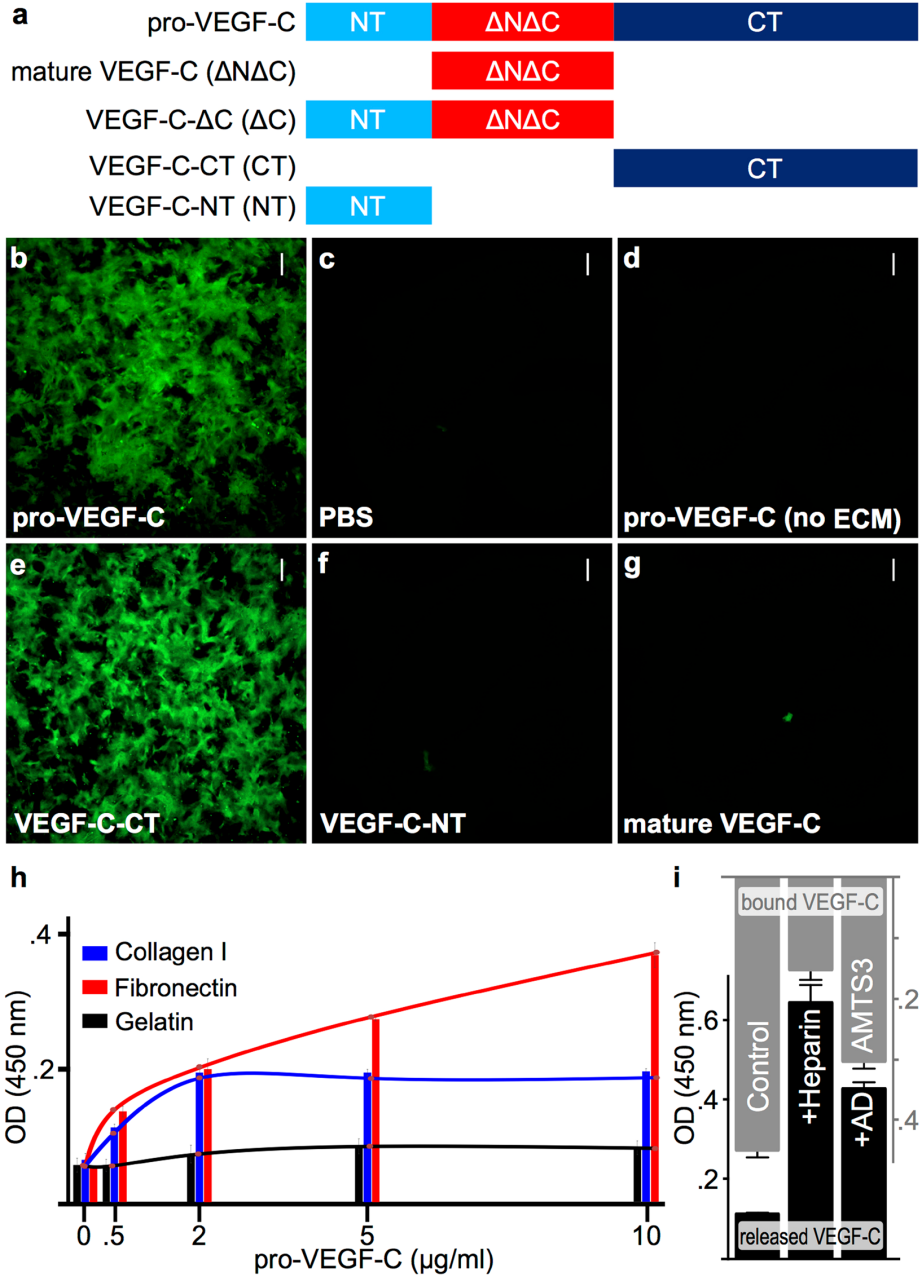
Pro-VEGF-C binds to extracellular matrix via its C-terminal domain. (**a**) Schematic representation of the domain structures of different VEGF-C forms used for protein production and the generation of transgenic mice. NT, N-terminal propeptide; ΔNΔC, mature VEGF-C (comprised largely of the VEGF homology domain) and CT, C-terminal propeptide. (**b–g**) After removal of cells and incubation with different recombinant proteins, ECM-bound VEGF-C was visualized with anti-VEGF-C antiserum 6 (which detects all forms of VEGF-C). Pro-VEGF-C (**b**) and the C-terminal domain of VEGF-C (**e**) bind to the deposited ECM whereas no binding could be detected for the N-terminal domain of VEGF-C (**f**) or the mature form of VEGF-C (**g**). PBS (**c**) and pro-VEGF-C applied to gelatinized coverslips without matrix deposition (**d**) were used as controls. (**h**) Pro-VEGF-C binds efficiently and specifically to fibronectin and, to a lesser extent to collagen I. Unlike collagen I, fibronectin appears to be able to bind additional pro-VEGF-C nonspecifically after saturation of the specific binding sites. (**i**) Solid phase binding assay of VEGF-C released from the cell free ECM. VEGF-C is efficiently released from ECM deposited by VEGF-C-expressing Cos-7 cells when incubated with recombinant ADAMTS3 or heparin, but only minor amounts of VEGF-C were released with D-MEM/0.1% BSA (Ctrl). Scale bars: 50 µm.

To study the association of VEGF-C with the ECM, we produced different forms and domain-deletion mutants of VEGF-C (schematically shown in Fig. 1a, Supplementary Fig. S1). Pro-VEGF-C bound to ECM deposited by NIH-3T3 fibroblasts (Fig. 1b), but not to an acellular control coverslip (Fig. 1d). The C-terminal domain of VEGF-C (VEGF-C-CT) exhibited a very similar binding to the ECM as pro-VEGF-C (Fig. 1e), whereas no or only very weak binding was detected for mature VEGF-C (Fig. 1g) or the N-terminal propeptide of VEGF-C (VEGF-C-NT, Fig. 1f). We obtained a similar result when matrix deposition occurred simultaneously with VEGF-C incorporation by VEGF-C-transfected Cos-7 cells (Supplementary Fig. S2b to f). Deposition of ECM by Cos-7 or NIH-3T3 cells was confirmed by immunostaining for fibronectin after decellularization (Supplementary Fig. S2g). In experiments with isolated proteins, binding of pro-VEGF-C was most efficient and concentration dependent to fibronectin, and to a lesser extent to collagen I (Fig. 1h). Furthermore, VEGF-C was released from the fibroblast-deposited matrix by addition of heparin or ADAMTS3 (Fig. 1i).

### Full lymphangiogenic potential of VEGF-C requires the C-terminal domain

Mutant zebrafish that express a truncated VEGF-C lacking the C-terminal domain (*vegfc* ^um18^) have lymphatic abnormalities, suggesting that the C-terminal domain has an important role during development of the lymphatic vasculature^5^. To study the lymphangiogenic potential of the C-terminal domain of VEGF-C, we generated transgenic mice which overexpress in the basal keratinocytes of the epidermis either the C-terminal domain of VEGF-C (VEGF-C-CT) or VEGF-C lacking the C-terminal domain (VEGF-C-ΔC), under the control of the keratin 14 (K14) promoter. The expression of both transgenes was confirmed by RT-PCR (Supplementary Fig. S3a) and by immunohistochemistry for the transgene-encoded proteins (Supplementary Fig. S3b).

**Figure 2:**
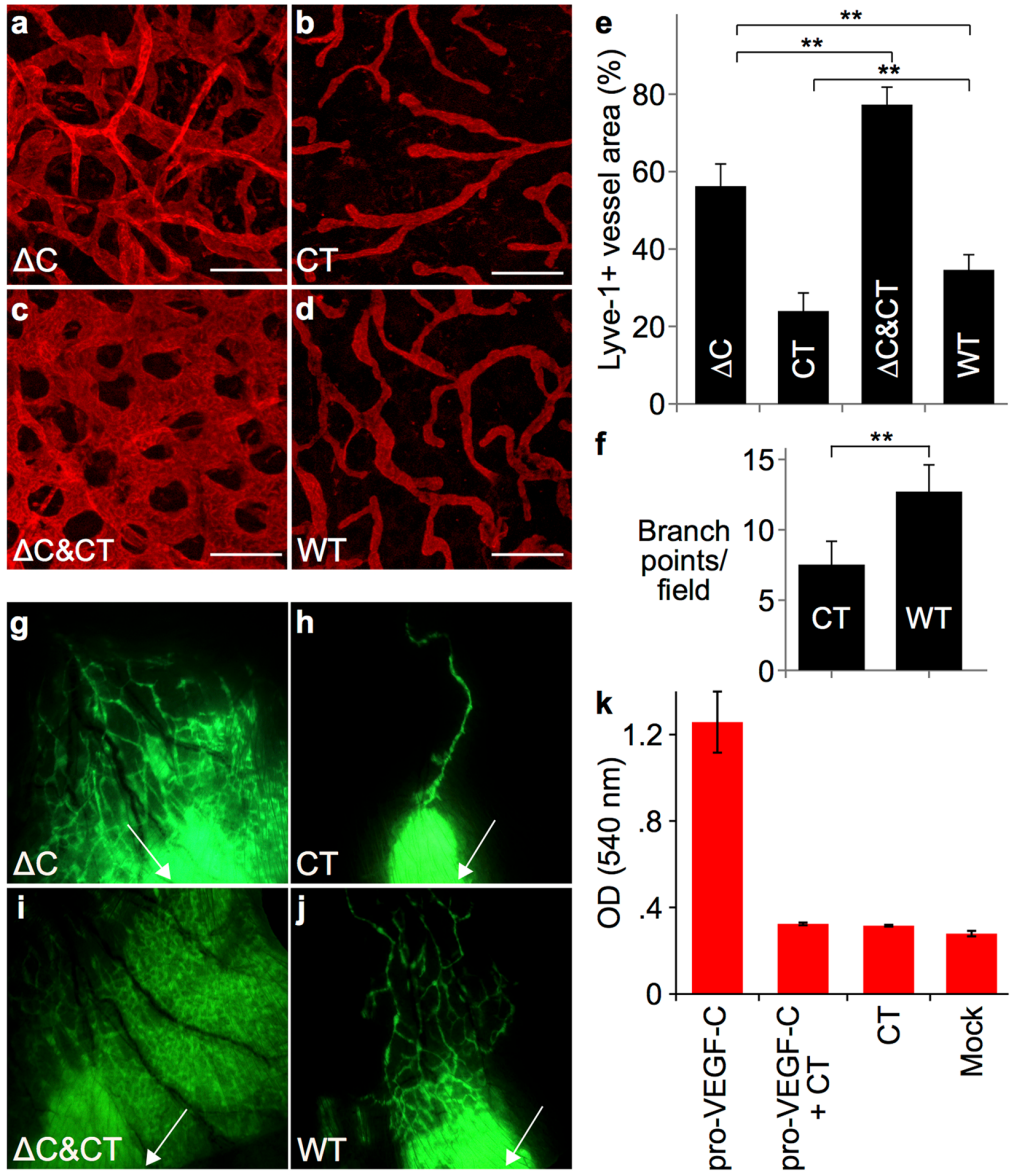
The C-terminus of VEGF-C enhances the lymphangiogenic response to VEGF-C, but represses lymphangiogenesis on its own. Lyve-1 whole-mount immunofluorescent stainings of ear skin from K14-VEGF-C-ΔC (**a**), K14-VEGF-C-CT (**b**), K14-VEGF-C-ΔC x K14-VEGF-C-CT (**c**) and wild type (WT) littermate (**d**) mice. (**e**) Quantification of Lyve-1-positive area in the whole-mount stained ear skin. **P < 0.01; n ≥ 4; Scale bar: 150 μm. (**f**) Quantification of branch points of Lyve-1-positive vessels per field. **P < 0.01; n ≥ 4. (**g**–**j**) Fluorescent microlymphangiography in the ears of adult mice. The injection site for FITC dextran is indicated by a white arrow. (**k**) Pro-VEGF-C (0.25 µg/ml) induces modest survival of Ba/F3-hVEGFR-3/EpoR cells, likely mediated by endogenous proteases. However, the addition of VEGF-C-CT (0.6 μg/ml, 10-fold molar excess) efficiently suppresses the survival mediated by pro-VEGF-C.

Lymphatic hyperplasia was apparent in the skin of the K14-VEGF-C-ΔC mice in comparison to their wild type littermates when analyzed by whole-mount Lyve-1 or Vegfr3 staining of the skin (Fig. 2a,d, quantification in Figs 2e and S4e, S4h), and by Lyve-1 immunohistochemistry of skin sections (Fig. S4a, S4d). The cutaneous lymphatic vessels in the K14-VEGF-C-ΔC mice were functional as determined by fluorescent microlymphangiography (Fig. 2g).

Surprisingly, there were less lymphatic capillaries in the skin of adult mice which overexpressed the C-terminal domain of VEGF-C than in the skin of their wild type littermates (Fig. 2b,d, quantification in Fig. 2e), and the lymphatic network had less branching points (Fig. 2b,d, quantification in Fig. 2f, Supplementary Fig. S4f, S4h). Fluorescent microlymphography showed that few of these lymphatic vessels were functional (Fig. 2h). However, the strongest lymphatic hyperplasia was observed in the skin of K14-VEGF-C-ΔC x K14-VEGF-C-CT double transgenic mice (Fig. 2c and Supplementary Fig. S4c, S4g, quantification in Fig. 2e). This was evident also in the fluorescent microlymphography (Fig. 2i). Almost complete inhibition of pro-VEGF-C activity by a 10-fold molar excess of the C-terminal domain of VEGF-C was also seen *in vitro* using a bioassay that measures Ba/F3 cell survival mediated by a chimeric VEGFR-3/EpoR receptor (Fig. 2k). The blood vasculature was not significantly affected in the skin of any of these transgenic mice (Supplementary Fig. S5a, S5b).

### The VEGF-C C-terminus rescues VEGF-C-ΔC activation and receptor binding *in vitro*

**Figure 3:**
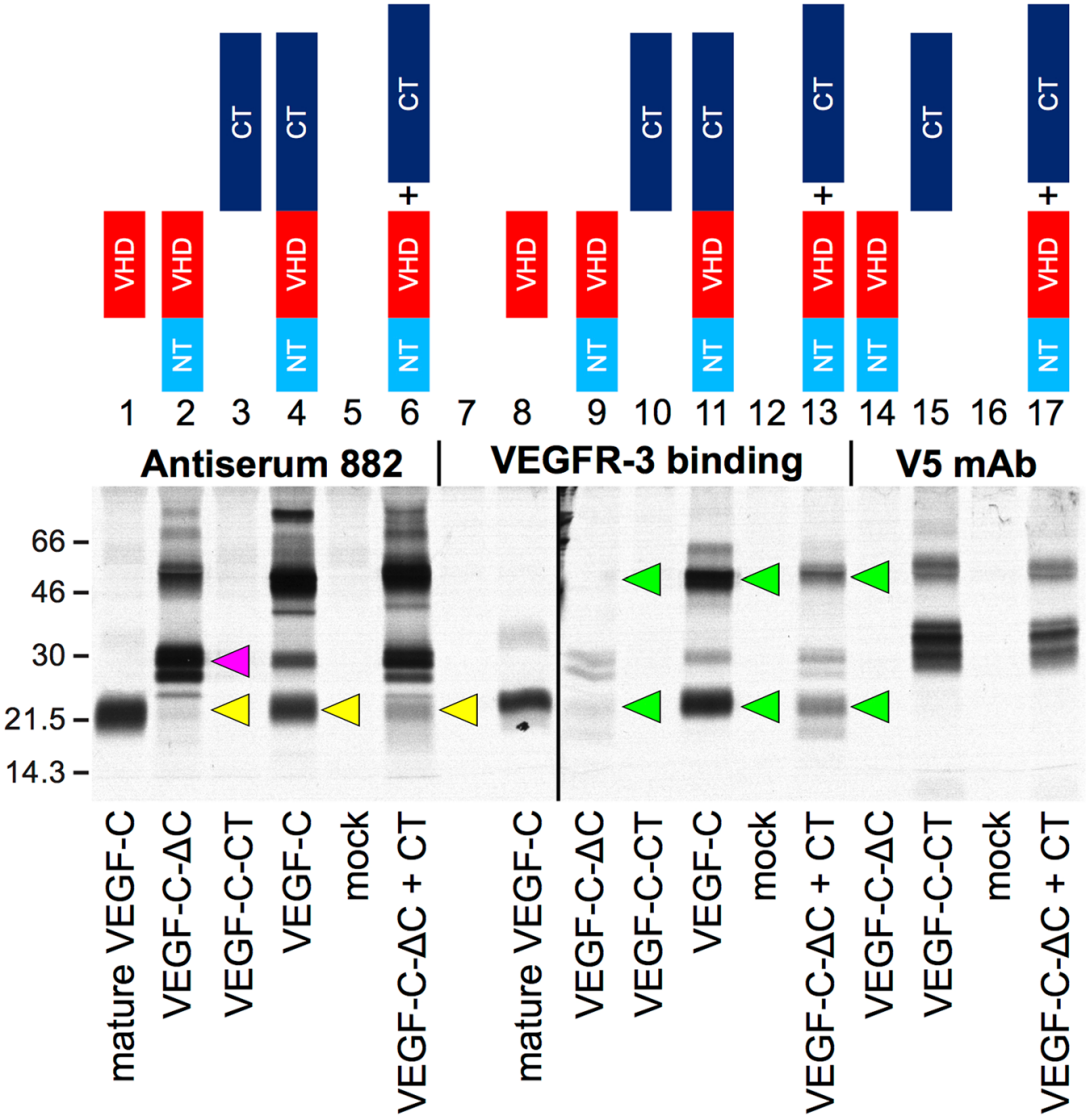
The VEGF-C C-terminus rescues proteolytic processing and receptor binding of VEGF-C- ΔC *in vitro*. Processing of pro-VEGF-C (magenta arrow) into mature VEGF-C (yellow arrows) and the ability to bind to VEGFR-3 (green arrows) are reduced when the C-terminus is omitted from VEGF-C. VEGF-C cleavage (lane 6) and its VEGFR-3 binding pattern (lane 13) are normalized when VEGF-C-ΔC is co-expressed with VEGF-C-CT. Metabolically labeled proteins were precipitated from the conditioned medium of transfected 293 T cells with VEGFR-3-Ig fusion proteins, with antiserum 882 or with anti-V5-antibody (VEGF-C-CT is V5-tagged) and analyzed by 12% SDS-PAGE under both reducing and non-reducing conditions. The full length Western blots are shown in Supplementary Fig. S12.

In order to understand why the C-terminal domain of VEGF-C enhanced lymphangiogenesis in the context of VEGF-C-ΔC but inhibited lymphangiogenesis when expressed alone, we studied the complementation of VEGF-C-CT and VEGF-C-ΔC *in vitro*. When VEGF-C was expressed from a full-length cDNA in 293 T cells, processing by the endogenously expressed proteases resulted in mature VEGF-C (Fig. 3, lane 4, yellow arrow)^4^. When the VEGF-C-ΔC truncation mutant, which lacks the C-terminal domain, was expressed, activation of VEGF-C was inhibited (lane 2, yellow arrow points to the strongly reduced band) and immature VEGF-C accumulated (lane 2, magenta arrow). However, when the C-terminal domain was co-expressed, the activation block was partially lifted and mature VEGF-C could be generated (lane 6, yellow arrow). Importantly, binding of both immature and mature VEGF-C to its receptor VEGFR-3 was rescued by the presence of the C-terminal domain (compare the bands indicated by the green arrows). This rescue occurred when the C-terminal domain of VEGF-C was expressed as part of the full-length VEGF-C polypeptide chain (lane 11) or as a separate polypeptide chain that complemented the VEGF-C-ΔC truncation mutation (lane 13). The same pattern was observed irrespectively of whether the proteins were resolved under non-reducing (Fig. 3) or reducing (Supplementary Fig. S6) conditions.

### CCBE1 and ADAMTS3 are located on the cell surface

Because the C-terminal domain of VEGF-C appeared necessary for VEGF-C activation, we turned to the CCBE1 and ADAMTS3 proteins. ADAMTS3 activates VEGF-C^16^, and CCBE1 is an obligate component of the activation complex^11^. Our previous observations had indicated that efficient and rapid VEGF-C activation occurs on cell surfaces, and thus, we hypothesized that ADAMTS3 and/or CCBE1 could affect VEGF-C distribution.

**Figure 4:**
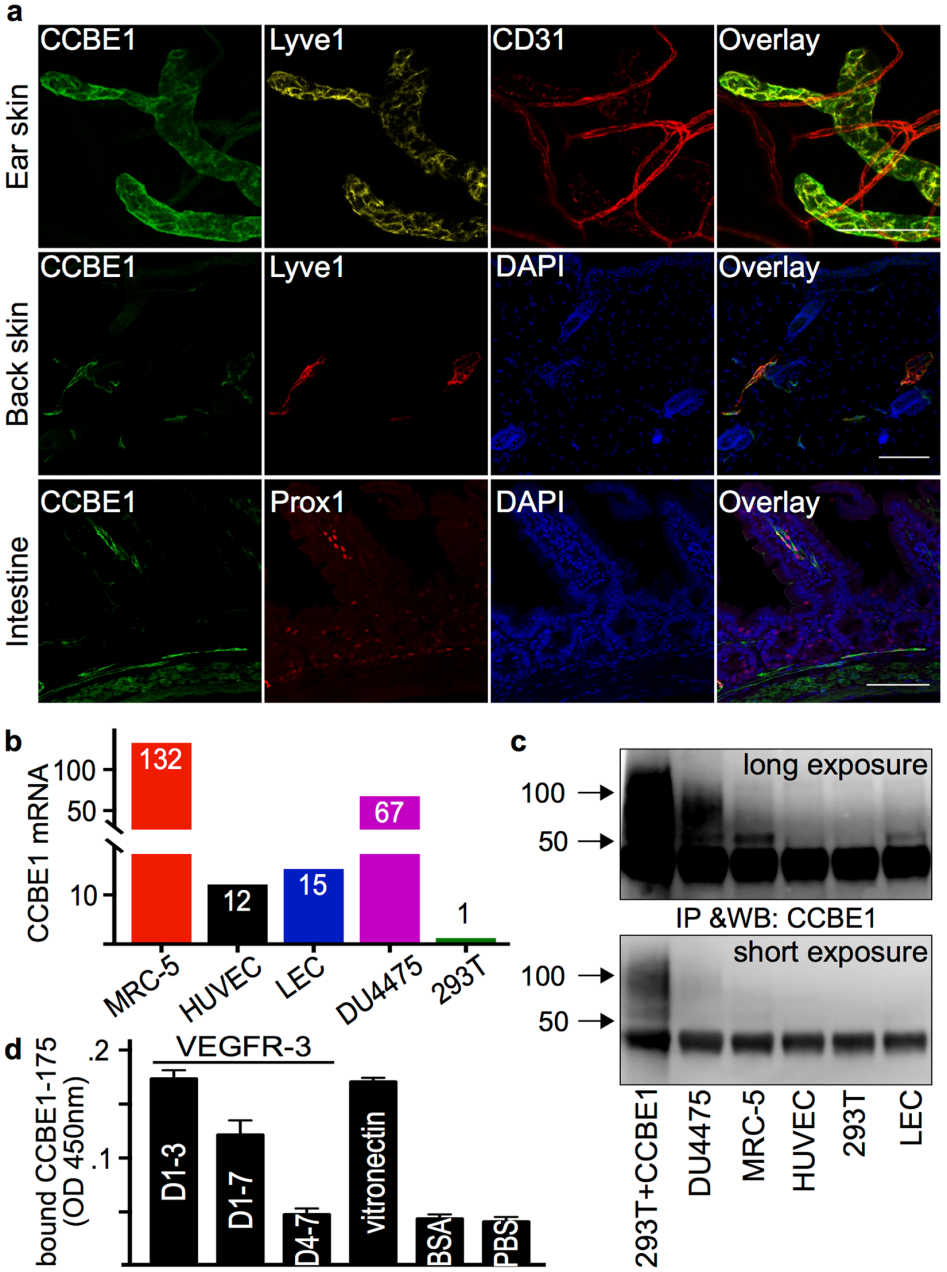
CCBE1 is expressed by fibroblasts and lymphatic endothelial cells, and localizes to lymphatic endothelial cell surface. (**a**) CCBE1 localization on the surface of lymphatic endothelial cells (LECs) in the mouse ear skin (whole mount staining), back skin and intestine (sections) as shown by co-staining with Prox-1 or Lyve-1. Expression of CCBE1 was analyzed by qPCR (**b**) and Western blotting (**c**). The full length blots are shown in the Supplementary Fig. S12. Among the primary cell lines analyzed, CCBE1 was expressed by LECs and HUVECs. From the tested cell lines, MRC-5 (fibroblast) and DU4475 (breast cancer) showed the highest expression of CCBE1. (**d**) In an assay with purified proteins, the N-terminal domain of CCBE1 (CCBE1-175) binds to immobilized VEGFR-3 and vitronectin. Binding to VEGFR-3 requires the presence of the VEGF-C binding domains of VEGFR-3 (immunoglobulin-like domains 1-3). Scale bars: 100 µm.

CCBE1 has been shown to be expressed by a subset of Prox-1 positive cells during early mouse embryonic development (E9.5)^19^ and by the Prox1 positive human skin LECs^20^. Transcriptomic analysis has revealed about three-fold higher expression levels of CCBE1 in LECs than in blood vascular endothelial cells (BECs)^21^. CCBE1 was assumed to localize to the ECM after secretion, apparently because of its affinity for vitronectin^12^. However, staining of mouse tissues with CCBE1, Prox-1, and Lyve-1 antibodies showed that CCBE1 is located primarily on the surface of LECs (Fig. 4a). Faint CCBE1 staining was also observed on BECs. CCBE1 was released from the surface of cultured LECs by high-salt treatment and detected by Western/ELISA (Supplementary Fig. S7d). Because also non-endothelial cells may produce CCBE1 *in vivo*, we performed qPCR (Fig. 4b, Supplementary Fig. S8) and Western blotting (Fig. 4c) of supernatants from cultured cells to identify cell types producing CCBE1. We found that endothelial cells, most notably LECs, expressed significant amounts of CCBE1. However, the highest expression levels were found in fibroblasts (e.g. in the lung fibroblast cell line MRC-5) and the human breast cancer cell line DU4475^22^. In solid-phase binding assays, we found that CCBE1 binds specifically to VEGFR-3 and that this binding was dependent on the presence of the first N-terminal Ig-like domains that form the VEGF-C/VEGF-D binding part of VEGFR-3 (Fig. 4d). When Cos-7 cells were transfected with expression vectors for CCBE1 and ADAMTS3, both ADAMTS3 and CCBE1 localized to the cell surface (Supplementary Fig. S7a, S7b). Similar to pro-VEGF-C, ADAMTS3 could be released from the cell surface by the addition of heparin (Supplementary Fig. S7b, S7c).

### The N-terminal domain of CCBE1 mediates pro-VEGF-C redistribution from the soluble phase to endothelial cell surfaces

**Figure 5:**
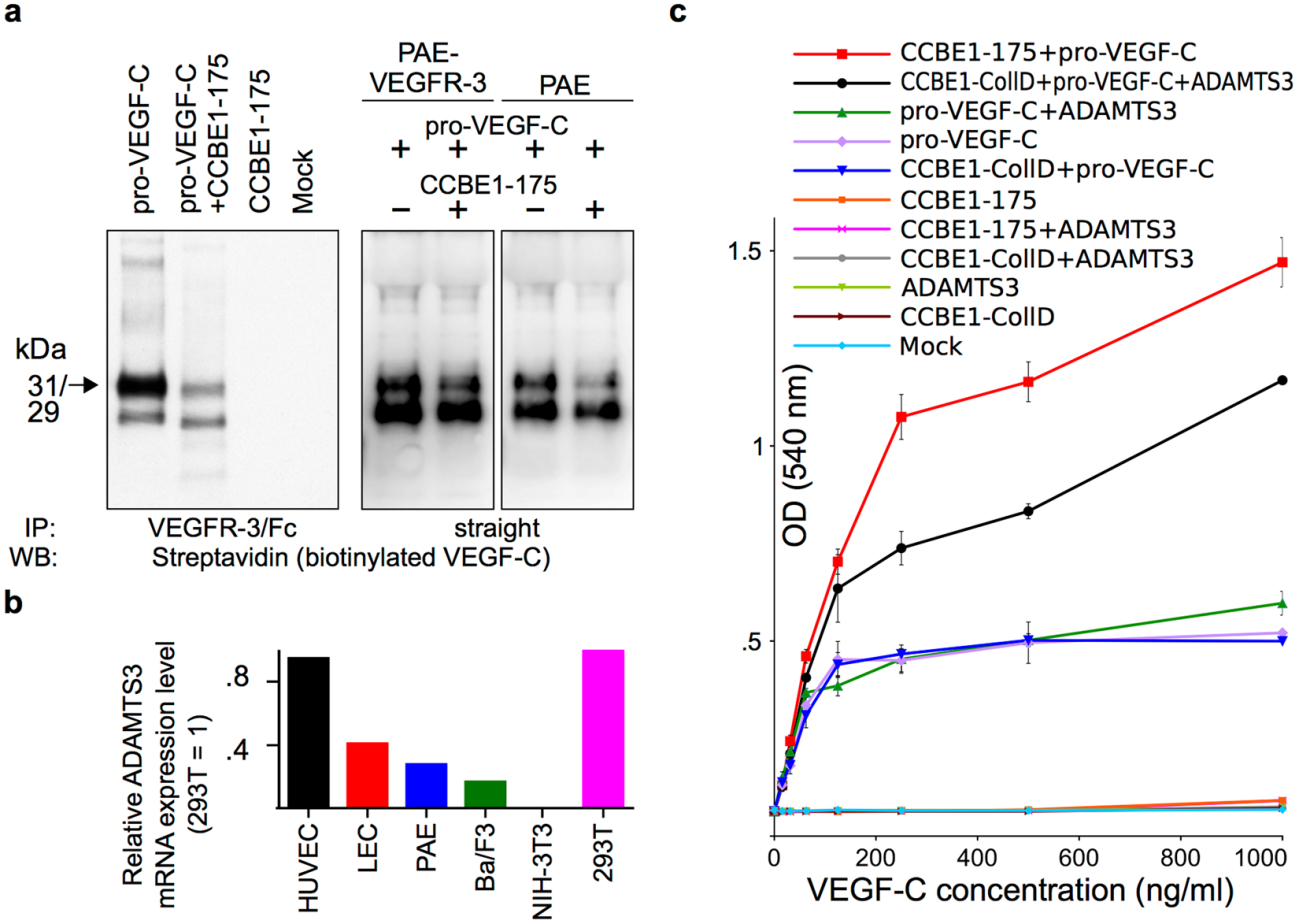
The N-terminal domain of CCBE1 affects the distribution and activity of pro-VEGF-C in cell-based assays. (**a**) VEGFR-3-expressing PAE cells were exposed to biotinylated pro-VEGF-C with and without the N-terminal domain of CCBE1 (CCBE1-175). Analysis of the supernatant after the incubation shows a marked reduction in the amount of pro-VEGF-C when incubated together with CCBE1-175. In the left panel, the leftover VEGF-C in the supernatant was immunoprecipitated with soluble VEGFR-3 receptor (VEGFR-3/Fc) before analysis. Note that only the upper band of pro-VEGF-C is subject to depletion. (**b**) Comparison of expression levels of ADAMTS3 by quantitative PCR. Expression levels of different cell types were normalized to 293 T cells. Note that all tested cell lines except for NIH-3T3 cells express ADAMTS3 to some degree. Two different primer pairs gave similar results (data is shown for primer pair 1). (**c**) Recombinant CCBE1-175 increases the effect of pro-VEGF-C on Ba/F3-hVEGFR-3/EpoR cells compared to pro-VEGF-C alone or a mixture of pro-VEGF-C and the C-terminal domain of CCBE1 (CCBE1-CollD). Increasing the amounts of ADAMTS3 by adding ADAMTS3-conditioned medium renders this assay also sensitive for the detection of CCBE1-CollD activity. Note that all five controls are superimposed. The full length blots are shown in Supplementary Fig. S13.

Since both pro-VEGF-C and CCBE1 interacted with VEGFR-3, we investigated how the interplay of pro-VEGF-C and CCBE1 influences pro-VEGF-C distribution. We stimulated VEGFR-3-expressing porcine aortic endothelial (PAE) cells with pro-VEGF-C with or without the N-terminal domain of CCBE1 (CCBE1-175). When stimulated in the presence of CCBE1-175, pro-VEGF-C concentration was decreased in the liquid phase (Fig. 5a, left panel). Notably, pro-VEGF-C concentration in the supernatant of PAE cells that do not express VEGFR-3 was also decreased, suggesting binding to HSPGs (Fig. 5a, right panel). To our surprise, assays with purified pro-VEGF-C, CCBE1, and VEGFR-3 showed that the N-terminal domain of CCBE1 competes with pro-VEGF-C for VEGFR-3 binding (Supplementary Fig. S9a).

### The N-terminal domain of CCBE1 promotes the effect of pro-VEGF-C

In order to study the effect of the N-terminal domain of CCBE1 on VEGFR-3 signaling, we wanted to identify assays that differentiate between the activities of the N-terminal and C-terminal domains of CCBE1. We first compared the expression of ADAMTS3 across a panel of cells by qPCR. We observed ADAMTS3 expression in HUVECs, LECs, Ba/F3-hVEGFR-3/EpoR, and in PAE cells stably expressing VEGFR-3 (PAE-VEGFR-3) (Fig. 5b). However, we did not detect any ADAMTS3 expression in NIH-3T3 fibroblasts. Because Ba/F3-hVEGFR-3/EpoR and PAE-VEGFR-3 cells expressed very little ADAMTS3, we chose these cells to assay the activity of the N-terminal domain of CCBE1. Notably, the N-terminal domain of CCBE1 (CCBE1-175) promoted robustly the survival of Ba/F3-hVEGFR-3/EpoR cells when applied together with pro-VEGF-C, whereas the application of the C-terminal domain of CCBE1 (CCBE1-CollD) together with pro-VEGF-C did not result in a detectable increase in the survival compared to pro-VEGF-C alone (Fig. 5c). To test if insufficient amounts of ADAMTS3 might be responsible for the lack of response towards CCBE1-CollD, we added 5 μl of ADAMTS3-conditioned medium. This boosted the activity of pro-VEGF-C in the presence of CCBE1-CollD to levels comparable to the activity of pro-VEGF-C in the presence of CCBE1-175. Furthermore, we stimulated PAE cells stably expressing VEGFR-3 with pro-VEGF-C, which resulted in very weak VEGFR-3 phosphorylation (Supplementary Fig. S9b, Lane 3,) compared to the phosphorylation induced by mature VEGF-C (Supplementary Fig. S9b, Lane 2). However, increased VEGFR-3 phosphorylation was seen when CCBE1-175 and pro-VEGF-C were used together (Supplementary Fig. S9b, Lane 5). In contrast, CCBE1-CollD induced only weak phosphorylation when incubated together with pro-VEGF-C (Supplementary Fig. S9b, Lane 7).

### The ADAMTS3 R565Q substitution interferes with CCBE1 interaction and cell surface association

**Figure 6:**
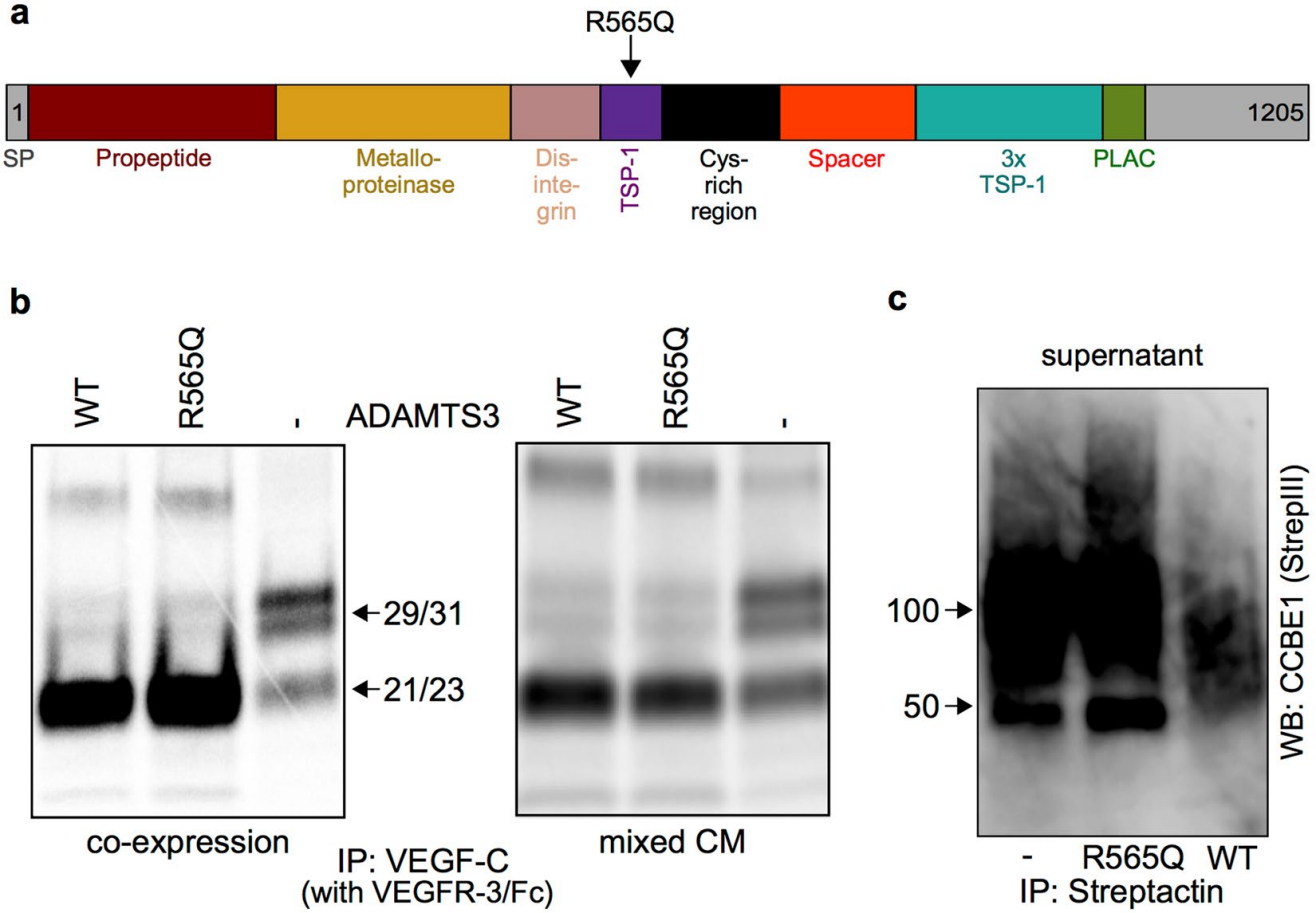
The R565Q substitution in ADAMTS3 interferes with the interaction of ADAMTS3 with CCBE1. (**a**) Schematic domain structure of ADAMTS3 (Uniprot) and location of the R565Q substitution. TSP-1: Thrombospondin 1; PLAC: Protease and lacunin. (**b**) The ADAMTS3 R565Q variant activates pro-VEGF-C equally well as wild type ADAMTS3 both when co-expressed in 293 T cells with VEGF-C (left) and when conditioned media (CM) from individually transfected 293 T cells were mixed (right). (**c**) Higher amounts of CCBE1 are present in the conditioned medium of ADAMTS3-R565Q variant or mock-transfected cells compared to the conditioned medium of cells transfected with wild type ADAMTS3. CCBE1 protein is secreted as a core protein of approximately 45–50 kDa and as a diffuse, chondroitinylated band around 100 kDa^11, 16^. The full length blots are shown in Supplementary Fig. S13.

We next tested the properties of an Adamts3 variant that has a heterozygous arginine 565 → glutamine (R565Q) missense substitution in the thrombospondin type 1 (TSP-1) motif of ADAMTS3 (Fig. 6a). This allele was originally identified as a rare heterozygous polymorphism in a lymphedema patient and in 6 unaffected members of the studied family as well as in 236 of the 120,650 alleles (0.1956%) in the Exome Aggregation Consortium (ExAC) database^23^. We first compared the effect of ADAMTS3 WT and the R565Q variant on pro-VEGF-C activation. Both ADAMTS3 forms supported processing of pro-VEGF-C when co-transfected into 293 T cells (Fig. 6b, left), or when conditioned supernatants were co-incubated (Fig. 6b, right). Since ADAMTS3 has been shown to interact with CCBE1 in previous studies^11, 16^, we wanted to see whether the R565Q substitution affects this interaction. By co-immunoprecipitation of conditioned media from 293 T cells transfected with CCBE1 and ADAMTS3, we detected a robust interaction between CCBE1 and wild type ADAMTS3, while the interaction between CCBE1 and the R565Q substitution appeared to be much weaker (Supplementary Fig. S10, lane 2 versus lane 3).

The decreased ability of the ADAMTS3 R565Q substitution to co-precipitate with CCBE1 was not due to lower levels of CCBE1 in the cell culture supernatant. On the contrary, the CCBE1 level in supernatants of ADAMTS3-R565Q-transfected cells was higher than in supernatants of wild type ADAMTS3-transfected cells (Fig. 6c, middle and right lane). The lack of interaction of the ADAMTS3 R565Q substitution with CCBE1 resulted in a shift from cell surface-bound to free CCBE1. This was evident as the mock-transfected cultures, which produce only low (endogenous) amounts of ADAMTS3, featured similar high levels of free CCBE1 as the cells transfected with the ADAMTS3 R565Q variant (Fig. 6c, left lane).

## Discussion

Our findings show that pro-VEGF-C becomes localized by virtue of its C-terminal domain to the extracellular matrix (ECM), notably fibronectin, and cell surfaces. Similarly, CCBE1 localizes to LEC surfaces via its N-terminal domain. Because the activation of pro-VEGF-C by ADAMTS3 is dependent on the assembly of a cleavage complex comprising CCBE1, ADAMTS3, and pro-VEGF-C^11^, the colocalization of these components at the cell surface largely increases the likelihood of VEGF-C activation, which is in line with previous observations that VEGF-C activation on the cell surface is enhanced more efficiently than in the soluble phase^16^ and that the activation can be enhanced by the N-terminal domain of CCBE1^12, 16, 24^.

The reduced lymphangiogenic potential of the C-terminally truncated VEGF-C in the transgenic mouse model likely results from the fact that efficient activation (i.e. N-terminal cleavage) of VEGF-C is dependent on the presence of its C-terminus as shown in the *in-vitro* complementation assays. By using a mutant that is resistant to C-terminal cleavage, Bui *et al*. showed that the N-terminal cleavage can occur independently from the C-terminal cleavage^11^. In both the wild type pro-VEGF-C and the cleavage-resistant mutant used by Bui *et al*., the C-terminus is still attached to the protein, whereas it is absent in the deletion mutant that we analyzed *in vivo* and *in vitro*. Hence, the presence of the (covalently or noncovalently linked) C-terminus appears to be necessary for efficient activation of VEGF-C by removal of its N-terminal propeptide. Overexpression of the C-terminal domain of VEGF-C in the transgenic mice reduced the number of lymphatic vessels compared to wild type littermates. In these mice, the C-terminal domain of VEGF-C likely inhibits endogenous VEGF-C activation by competing with the C-terminal domain in pro-VEGF-C for the assembly of the cleavage complex. Such inhibition could also be demonstrated in the cell-based Ba/F3-VEGFR-3/EpoR assay (Fig. 2k).

The N-terminal domain of CCBE1 facilitated the immobilization of pro-VEGF-C from the soluble phase to the surface of endothelial cells, independently of the presence of VEGFR-3. In contrast, in the assays with purified proteins, pro-VEGF-C competed with CCBE1 for direct binding to VEGFR-3. However, the net effect of CCBE1 on the cell surface increased local VEGF-C concentration, which is also promoted by neuropilin-2^16^, syndecan-4^18^, and β-1 integrin^25, 26^, which stabilize the VEGFR-3/VEGF-C interaction. In addition to direct activation of VEGFR-3-bound pro-VEGF-C^16^, a significant proportion of pro-VEGF-C could first interact with CCBE1 and ADAMTS3 independently of VEGFR-3 on cell surface sites containing syndecan-4^18^, and after cleavage, the mature VEGF-C would activate adjacent VEGFR-3.

The presence of the CCBE1/ADAMTS3 activation complex may determine if VEGF-C forms a growth factor gradient or acts in a non-directional fashion. Activating pro-VEGF-C on the surface of LECs would provide mainly a nondirectional, mitogenic signal, while the generation of mature VEGF-C from ECM-sequestered pro-VEGF-C could provide an instructional gradient for network formation and patterning, similar to how VEGF-A gradients act in angiogenesis^27, 28^. Such instructional signal appears to be missing in mice that lack the EGF-like domains of CCBE1. In these mice, LECs can form clusters but cannot organize into a functional network^15^, which is very different from mice that lack the collagen-like domain of CCBE1 and phenotypically mimic the full *Ccbe1* gene deletion. During development, ECM-release of VEGF-C is likely mediated exclusively by ADAMTS3 as the *Adamts3* gene deletion halts all lymphangiogenesis^17^. However, during tissue repair and pathological lymphangiogenesis, other proteases, such as plasmin, are probably involved^29^.

In the VEGFR-3 phosphorylation assays and in the Ba/F3-hVEGFR-3/EpoR assays, the C-terminal domain of CCBE1 failed to show significant activity, whereas the N-terminal domain of CCBE1 enhanced VEGFR-3 phosphorylation by VEGF-C and stimulated cell survival. The low amounts of ADAMTS3 expressed by PAE and Ba/F3 cells appeared to discriminate against the cleavage-enhancing function of the ﻿C﻿-terminal domain of CCBE1. When ADAMTS3 amounts are limited, its colocalization with VEGF-C and CCBE1 appears to be essential for significant VEGF-C activation. Addition of exogenous ADAMTS3 allowed the detection of the ﻿activity of ﻿the ﻿C-terminal domain of CCBE1 in the VEGF-C activation assay. Interestingly, addition of CCBE1-CollD together with pro-VEGF-C did not increase the activation of pro-VEGF-C above the background levels. A complete absence of ADAMTS3 precludes the detection on the activity of all forms of CCBE1. Indeed, pro-VEGF-C stimulation of NIH-3T3/hVEGFR-3 cells, which have no detectable ADAMTS3, repeatedly failed to increase VEGFR-3 phosphorylation, irrespective of whether the N-terminal or C-terminal domain of CCBE1 was used (unpublished data of the author).

The presence of the TSP-1 motif in ADAMTS3 suggested its association with cell surfaces, which was confirmed by our Western blotting data. This would additionally promote the preferential assembly of the cleavage complex on cell surfaces. The TSP-1 motif of ADAMTS4 has been reported to bind to its cell surface interaction partner CD36^30^. We did not observe any differences in the processing of VEGF-C between 293 T cells transfected with VEGF-C and wild type ADAMTS3 or the ADAMTS3-R565Q mutant vector, but CCBE1 failed to associate with the cell surface in the ADAMTS3-R565Q-transfected cells.

The R565 residue is conserved among all ADAMTS family members and between species (Supplementary Fig. S11). The R368H mutation of the homologous amino acid in ADAMTS13, which cleaves von Willebrand factor, constituted one allele of a compound heterozygous form of congenital thrombotic thrombocytopenic purpura^31^. So far, all *in-vitro* analyses of the ADAMTS13 mutant have assayed only effects on synthesis, secretion or catalytic activity. While we have not compared the enzyme activity or secretion of the R565Q mutant and wild type proteins, our data suggests an altered extracellular localization as a potential disease mechanism. Since the ADAMTS3 R565Q allele was heterozygous in the patient and since it did not completely abolish the interaction of ADAMTS3 with CCBE1, it may not alone explain the lymphedema phenotype, suggesting that it acts as a modifier of the lymphedema phenotype in this family.

**Figure 7:**
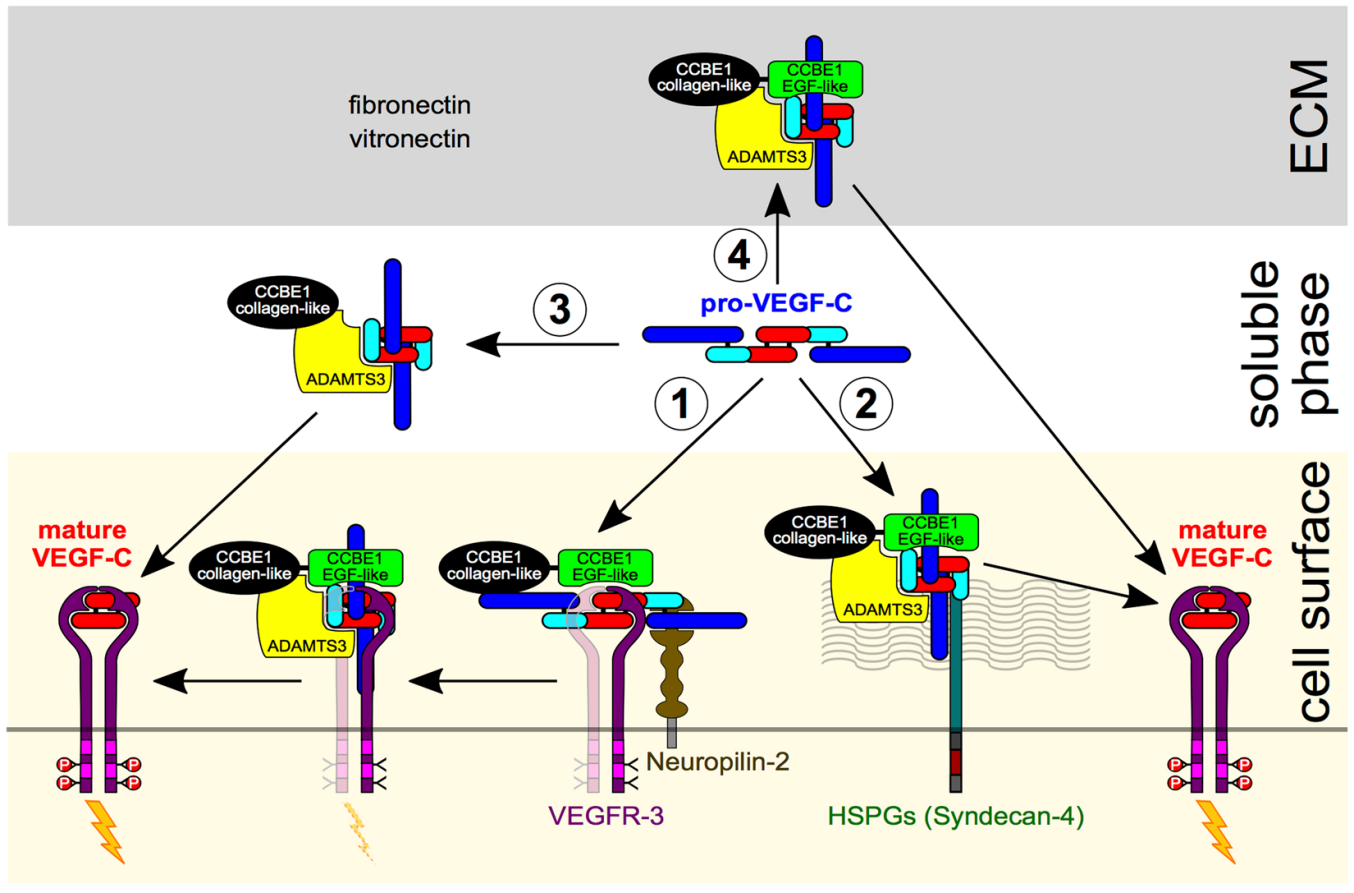
Schematic view of VEGF-C activation based on current experimental evidence. Proteolytic cleavage of pro-VEGF-C simultaneously activates and mobilizes VEGF-C. We propose four different modes of VEGF-C activation: 1. Activation of VEGFR-3-bound VEGF-C^16^; 2. Activation of HSPG-bound VEGF-C^18^; 3. Activation of VEGF-C in the soluble phase^11^; and 4. Activation of ECM-bound VEGF-C. After proteolytic activation, VEGFR-3-bound VEGF-C can immediately start signaling (activation mode #1), while HSPG-bound VEGF-C^18^ first needs to translocate to VEGFR-3 (activation mode #2). Although activation of VEGF-C does happen in solution (activation mode #3), the localization of pro-VEGF-C, CCBE1 and ADAMTS3 indicates that a significant fraction of the VEGF-C activation is associated with the ECM (activation mode #4) or cell surfaces (activation modes #1 and #2). In the activation mode #2, pro-VEGF-C is shown to be processed while HSPGs-attached. However, pro-VEGF-C might as well translocate first from HSPGs to VEGFR-3 and become activated while VEGFR-3-bound. The role of CCBE1 is twofold: It accelerates the proteolytic cleavage (mediated by its C-terminal domain) and localizes pro-VEGF-C to efficiently form the trimeric activation complex (mediated by its N-terminal domain).

Based on our present study and the results of Bui, Johns and Roukens^11, 15, 18^, we thus suggest a refined model of VEGF-C activation (Fig. 7). According to our model, VEGF-C and CCBE1^12^ are sequestered in the ECM and on cell surface. To initiate lymphangiogenesis, pro-VEGF-C is first mobilized. A protease-mobilized VEGF-C would immediately be available for signaling, whereas pro-VEGF-C could either be activated in the extracellular space or translocate to the endothelial cell surface, where it can be efficiently activated by a CCBE1/protease complex. Depending on whether this cleavage complex assembles on VEGFR-3 or HSPGs, VEGF-C could start signaling immediately or would first require a translocation from the HSPG to VEGFR-3.

We speculate that the distinct locations of VEGF-C activation could determine the migration versus proliferation promoting effects of VEGF-C. Mature VEGF-C has less affinity for heparin than pro-VEGF-C^18^. Its release from ECM deposits could be instrumental for gradient formation, thus directing and organizing the lymphangiogenic response, while activation of pro-VEGF-C on lymphatic endothelial cell surface would not provide a growth factor gradient for migration, thus serving only as a signal for LEC proliferation and survival.

Delineating VEGF-C activation is not only important in order to understand the development of the lymphatic system, but also to understand tumor angiogenesis, because mature VEGF-C is also able to activate the VEGFR-2 signaling pathway and because some tumors might escape antiangiogenic therapy by employing VEGF-C instead of VEGF-A^32^. It remains to be elucidated if the inhibition of VEGF-C activation can provide a treatment strategy for such tumors.

## Materials and Methods

### Transfections, Metabolic Labeling, Immunoprecipitation, SDS-PAGE, Western Blotting and Protein Analysis

293 T and Cos-7 cells transfections and procedures were performed as described^16^.

### Ba/F3-VEGFR/EpoR Assays

The Ba/F3-hVEGFR-3/EpoR^33^ bioassay was performed with conditioned cell culture medium or recombinant proteins as described^34^. For the VEGF-C inhibition assay, 0.25 μg/ml of pro-VEGF-C was mixed with 0.6 μg/ml (a 10-fold molar excess) of VEGF-C-CT. Similarly, for studying the CCBE1-175-mediated effect of VEGF-C, CCBE1-175 and CCBE1-CollD were used at concentrations of 10 μg/ml, and pro-VEGF-C at a concentration of 1 μg/ml. 293 T cells were transfected with plasmid encoding ADAMTS3 and 5 μl of the conditioned media was used per well for the assay.

### Stimulation of VEGFR-3 Phosphorylation

Stimulation of VEGFR-3 phosphorylation was performed in 60-mm cell culture dishes (~1 Mio. cells/dish) as described^16^, with ΔNΔC-VEGF-C, pro-VEGF-C, the N-terminal domain of CCBE1 (CCBE1-175), and the C-terminal domain of CCBE1 (CCBE1-CollD) at concentrations of 0.02, 0.4, 5 and 5 μg/ml, respectively, in 0.5 to 1 ml D-MEM/0.1% BSA.

### *In-vitro* complementation assay

293 T cells were transfected with plasmids encoding full-length VEGF-C, VEGF-C-ΔC, VEGF-C-CT, both VEGF-C-ΔC and VEGF-C-CT, or with an empty plasmid and metabolically labeled with [^35^S]-cysteine/[^35^S]-methionine (PerkinElmer, Waltham, MA). After 48 hours, supernatants were harvested and immunoprecipitated proteins were separated by SDS-PAGE and visualized by autoradiography.

### Cloning, protein production and purification

For details see Supplementary Information.

### Generation of transgenic mice

The K14-VEGF-C-ΔC and K14-VEGF-C-CT expression cassettes were injected into fertilized FVB/N mouse oocytes. The mice were genotyped by PCR of tail DNA using forward primer 5′-GCTCTGGGTTCCAGGTTCCACTGG-3′ and reverse primers 5′-CGTCTTGCTGAGGTAGCTCGTGC-3′ (for K14-VEGF-C-ΔC) or 5′-CGTAGAATCGAGACCGAGGAGAGG-3′ (for K14-VEGF-C-CT). Transgene expression was confirmed by RT-PCR of embryonic skin RNA using the genotyping primers. Three founder lines from both transgenic strains were selected for further analysis. The National Board for Animal Experiments of the Provincial State Office of Southern Finland approved all animal experiments carried out in this study and all animal experiments were performed according to the guidelines and regulations. Mice were housed in individually ventilated cages with enrichment materials in a facility monitored by the Federation of European Laboratory Animal Science Associations guidelines and recommendations.

### Antibodies

Anti-VEGF-C antiserum 6^35^, anti-VEGF-C antiserum 882^4^, anti-VEGF-C antibodies (R&D Systems, Minneapolis, MN, AF752), anti-V5 antibody (Invitrogen, Carlsbad, CA, #46-0705), anti-phosphotyrosine antibody 4G10 (Merck Millipore, Billerica, MA), anti-CCBE1 antibodies (Atlas Antibodies AB, Stockholm, Sweden, #HPA041374) and anti-ADAMTS3 antibodies (Santa Cruz, Dallas, TX, sc-21486) were used for both immunoprecipitation and Western blotting. Anti-VEGFR-3 antibodies (Santa Cruz, sc-321), chimeric VEGFR-3/Fc^33^ and streptactin sepharose (IBA, Göttingen, Germany) were used for immunoprecipitation only. Streptactin-HRP conjugate (IBA, #2-1502-001) was used for Western blots. The following antibodies were used for staining; polyclonal rabbit anti-mouse Lyve-1^13^, goat anti–human PROX1 (R&D Systems, AF2727), anti-Fibronectin (SIGMA, F3648), anti-VEGF-C antiserum 6, anti-CCBE1, anti-podocalyxin (R&D Systems, AF1556) and goat anti-mouse VEGFR-3 (R&D Systems, AF743). The primary antibodies were detected with the appropriate Alexa Fluor 488, 594, or 647 secondary antibody conjugates (Molecular Probes).

### Analysis of lymphatic and blood vessels

Whole mount staining was performed as described^36^ with Lyve-1, CCBE1 and VEGFR-3 antibodies, followed by Alexa-conjugated secondary antibodies 488, 594 and 647 (Molecular Probes, Invitrogen, Life Technologies, Carlsbad, CA).

Paraffin sections were immunostained with Lyve-1 antibodies using the tyramide signal amplification kit (NEN Life Sciences/PerkinElmer Life and Analytical Sciences, Boston, MA). Podocalyxin antibodies were used for the immunofluorescence staining of vessels.

The area covered by lymphatic vessels (photomicrographs of Lyve-1-stained whole mount sections, 6 photomicrographs/mouse) and the number of podocalyxin-positive vessels were quantified using the ImageJ software. The lymphatic vessel branch points per field were counted manually. For quantification, 4–6 mice were used for each genotype. For the visualization of functional lymphatic vessels, FITC-conjugated dextran (2000 kDa, Sigma-Aldrich, St. Louis, MO) was injected intradermally into the ear and the uptake of the dye by lymphatic vessels was visualized by fluorescence microscopy.

For the detection of CCBE1 in lymphatic vessels, tissue samples (back skin and intestine) were obtained from 7–8 weeks old C57BL/6 J mice. Immunofluorescence staining was performed on paraffin sections using antibodies against CCBE1, Lyve-1, Prox-1 and podocalyxin and corresponding Alexa-conjugated secondary antibodies (Molecular Probes).

Samples were imaged using AxioImager.Z2 upright epifluorescence microscope and Zeiss LSM 780 confocal microscope.

### ECM binding studies of VEGF-C

NIH-3T3 or Cos-7 cells were grown on 0.1% gelatin-coated coverslips in 24- or 48-well culture plates for up to 8 days, changing medium (supplemented with ascorbic acid, 50 μg/ml, SIGMA) every 48 hours. The cells were gently removed as described^12^ or alternatively by incubating cells with 20 mM NH_4_OH, 5 mM EDTA at RT with gentle swirling of the plate so that the intact ECM remained on the coverslips. ECM was incubated with 4 μg/ml of pro-VEGF-C or 4-fold molar excess of other VEGF-C proteins overnight at 4 °C followed by fixation with 4% PFA and blocking in 1% BSA in PBS for 1 hour at RT. The cover slips were then stained by immunohistochemistry using anti-VEGF-C antiserum 6 (1:200) and anti-fibronectin (1:200) followed by Alexa 488 conjugated secondary antibody. Fluorescent images were obtained with an AxioImager.Z2 upright epifluorescence microscope (Carl Zeiss AG, Oberkochen, Germany).

### Cell culture and generation of stable cell lines

293 T, 293 F, Cos-7, Mouse embryonic fibroblast (MEF, a gift from Tomi Mäkelä, University of Helsinki), 293T-CCBE1-StrepIII^16^, PAE-VEGFR-3^37^, Ba/F3-hVEGFR-3/EpoR^33^ and NIH-3T3 cells were grown in D-MEM 10% FCS. MRC-5 cells were grown in E-MEM 10% FCS. LECs and HUVECs were purchased from Promocell (Heidelberg, Germany) and maintained per the instructions of the supplier.

### ADAMTS3-R565Q/CCBE1 co-immunoprecipitation

293T-CCBE1-StrepIII cells were transfected with ADAMTS3, ADAMST3-R565Q or mock expression constructs. After 24 hours, the media were changed to D-MEM/0.2% BSA. After 48 hours, ADAMTS3 was immunoprecipitated from the conditioned media with antibodies/Protein G sepharose or streptactin sepharose. The immunoprecipitates were then separated by SDS-PAGE and detected using streptactin-HRP.

### Solid-phase binding assays

Solid-phase binding assays were performed to determine the relative binding of CCBE1-175 to VEGFR-3. 96-well microtiter plates (Maxisorp, Nunc) were coated with recombinant vitronectin-Fc^38^, different human VEGFR-3/IgG fusion proteins or BSA at 20 μg/ml in PBS overnight at 4 °C. Plates were then extensively washed with PBS and blocked with 2% BSA for 2 hours at RT. CCBE1-175-V5 (10 μg/ml in PBS) was allowed to bind to the coated plates overnight at 4 °C in the presence of 1 mM CaCl_2_. Plates were washed with 0.05% Tween-20/PBS (PBST), blocked with 5% BSA/PBST (PBSTB) for 1 hour at RT and then incubated for 2 hours with anti-V5 antibody (1:500 in PBSTB). Plates were washed with PBST and then incubated with anti-mouse-HRP (1:500 in PBSTB) for 1 hour at RT. After extensive washing with PBS, plates were incubated in TMB (Thermo Scientific) for 7 minutes followed by addition of 1 M HCl to stop the reaction. The plates were read at 450 nm.

To quantitate pro-VEGF-C binding to matrix proteins, collagen type 1 (BD Biosciences, #354249), gelatin (SIGMA, G1890) or fibronectin (SIGMA, F08952) were used for coating (in PBS at 20 μg/ml). Anti-VEGF-C antiserum 6 (1:2000) was used to detect VEGF-C.

### pro-VEGF-C release assay

The pro-VEGF-C release assay from ECM was performed by transfecting Cos-7 cells with plasmids encoding full length VEGF-C in 96-well-plates. 24 hours post transfection, the cell culture medium was changed to D-MEM/0.2% BSA, and 72 hours post transfection the medium was removed and wells were rinsed with PBS, followed by decellularization with 20 mM NH_4_OH, 5 mM EDTA in PBS for 10 mins at RT. Wells were washed with PBS and incubated with 5 μg/ml of ADAMTS3 or 100 μg/ml of heparin in D-MEM/0.2% BSA for 2 hours at 37 °C. After incubation, VEGF-C amounts were quantified separately for the leftover VEGF-C in the cell-free matrix (on the same plate) and the released VEGF-C in the supernatant (on a separate 96-well Maxisorp plate after overnight immobilization). Washing, blocking and detection were performed as for the solid phase binding assays.

### mRNA expression analysis

mRNA expression was performed as described^16^. For details see Supplementary Information.

### Protein analysis in lymphatic endothelial cells

To detect CCBE1 protein in the supernatants of primary cells and cell lines, equal amount of cells were plated on 10 cm dishes. 24 hours later, the medium was changed to D-MEM/1% FCS and cells were incubated further for 48 hours. Supernatants were harvested and CCBE1 was immunoprecipitated, resolved via SDS-PAGE and visualized by Western blotting.

### Statistical analysis

Data were analyzed using GraphPad Prism statistical analysis software (Version 6). Data are represented as mean ± SD. Significance of the differences was determined using one-way ANOVA followed by Tukey posthoc test. All statistical tests were two-tailed. When comparing two means, unpaired Student’s t-test was used. Data were considered significant at P < 0.05.

## Electronic supplementary material

Supplementary Information
