# Supplementary material for: Efficient activation of the lymphangiogenic growth factor VEGF-C requires the C-terminal domain of VEGF-C and the N-terminal domain of CCBE1
Source: Sci Rep. 2017 Jul 7;7:4916. doi: 10.1038/s41598-017-04982-1 (PMC5501841; doi:10.1038/s41598-017-04982-1)
Supplement: Supplementary file 1 — Supplementary Information [file 41598_2017_4982_MOESM1_ESM.pdf]

SUPPLEMENTARY INFORMATION

Efficient activation of the lymphangiogenic growth factor VEGF-C requires the C-terminal domain of VEGF-C and the N-terminal domain of CCBE1

Sawan Kumar Jha, MSc[1]; Khushbu Rauniyar, MSc[1]; Terhi Karpanen, PhD[4]; Veli-Matti Leppänen[1,2], PhD; Pascal Brouillard, PhD[5]; Miikka Vikkula, MD, PhD[5,6]; Kari Alitalo, MD, PhD[1,2,3]; Michael Jeltsch, PhD[1,2*]

[1]Translational Cancer Biology Research Program, [2]Wihuri Research Institute, Biomedicum Helsinki, University of Helsinki, Finland

[3]Helsinki University Central Hospital, Helsinki, Finland

[4]University Hospital Radiumhospitalet and K. G. Jebsen Center for Cancer Immunotherapy, Institute for Clinical Medicine, University of Oslo, Oslo, Norway

[5]de Duve Institute, University of Louvain, Brussels, Belgium

[6]Walloon Excellence in Life Sciences and Biotechnology (WELBIO), University of Louvain, Brussels, Belgium

*Author for correspondence: Dr. Michael Jeltsch, Translational Cancer Biology Research Program and Wihuri Research Institute, Biomedicum Helsinki, P.O.B. 63 (Haartmaninkatu 8), 00014 University of Helsinki, Finland, Phone: +358-2941-25514, Fax: +358-2941-25510, E-mail: michael@jeltsch.org

SUPPLEMENTARY METHODS

Cloning

The cDNAs coding for VEGF-CΔC or VEGF-C-CT with the IgK signal peptide were PCR amplified from VEGF-C-ΔC/pMosaic or VEGF-C-CT/pMosaic plasmid[1] using the forward primer 5'-GCGATATCCCGCCGCCACCATGGAGACAGACACACTCCTGC-3' and the reverse primers 5'-GCTCTAGATTAACGTCTAATAATGGAATG-3' or 5'-GCTCTAGATTACGTAGAATCGAGACCGAGGAGAGGGTTAGGGATAGGCTTACCGCTCATTTGTGGTCTTTTCC-3' (containing sequences encoding a V5-tag), respectively, and cloned into the K14 expression vector[2].

The pCI-Neo-hADAMTS3-R565Q-V5-H6 mutant was created by amplifying pCI-Neo-hADAMTS3-V5-H6[3] using primers 5'-GTTCAATTGGGGGTCATGGACTAAATTTGGCTCCTGTTCTCAGACATGTGGAAC-3' and 5'-ATGAATGCAATTGTTGTTGTTAACTTGTTTATTGC-3'. The amplified product was then inserted into pCI-Neo-hADAMTS3-V5-H6 using *MfeI* restriction sites.

Protein production and purification

VEGF-C, ADAMTS3, VEGF-C-CT-V5, VEGF-C-NT-Fc and CCBE1-175 proteins were produced and purified as described[3]. Similarly, human VEGFR-3/IgG D1-3, D1-7 and D4-7 were purified as described[4].

For purification of the C-terminal domain of human CCBE1 (CCBE1-CollD), S2 cells were transfected with constructs encoding the C-terminus of human CCBE1 (amino acid residues

207 to 406 followed by a hexahistidine tag) using Effectene (Qiagen, Venlo, The Netherlands). 48 hours post transfection, cell pools were selected with 400 μg/ml hygromycin for 3-4 weeks. Cells were grown in suspension culture and induced for 3-4 days with 1 mM $CuSO_4$. The pH of the conditioned medium was adjusted to 8 and the NaCl concentration 450 mM followed by batch-binding to Ni^{2+} NTA sepharose (Ni Sepharose High Performance, GE Healthcare, #310563) with gentle rotation at 4°C overnight. The Ni^{2+} NTA sepharose was loaded onto a chromatography column and washed with 20mM imidazole. The elution was performed with 250mM imidazole and the major elution fraction was gel filtrated on a Superdex 200 column using phosphate buffered saline (450 mM NaCl, pH 7.4).

mRNA expression analysis

Total RNA was isolated from MRC-5, HUVECs, LECs, DU4475, 293T, MEF, NIH-3T3, PAE-hVEGFR3 and Ba/F-hVEGFR-3/EpoR cells with NucleoSpin RNA II kit according to the manufacturer's protocol (Macherey-Nagel, Düren, Germany). cDNA was synthesized using iScript cDNA synthase kit (Bio-Rad; Hercules, CA) and qPCR was carried out using SYBR green chemistry using the Bio-Rad CFX96 Real-Time System. All data were normalized to HPRT (Hypoxanthine Phosphoribosyltransferase) and quantification was performed using the 2-DDCT method. The primers used were: hCCBE1 (fwd 5'-CCCCTGGTTCTTTCGACTT-3', rev 5'-TAGCTGGGAAATTCCTGAGGTAAAG-3') and hHPRT (fwd 5'-TGAGGATTTGGAAAGGGTGT-3', rev 5'-TCCCCTGTTGACTGGTCATT-3').

For ADAMTS3 mRNA expression in PAE-VEGFR-3, Ba/F-hVEGFR-3/EpoR, NIH-3T3,

LECs, HUVECs and 293T cells, primers were used that could recognize ADAMTS3 and HPRT in three species (mouse, human and pig): ADAMTS3-primer pair 1 (fwd 5’-TTCCAGGAACCTCTGTTGCC-3’, rev 5’-GCTGATCTCTTGTAGACAAC-3’), ADAMTS3-primer pair 2 (fwd 5’-CGTTTCCATGGCAAAGAGC-3’, rev 5’-GCACTCCGAGGGACTCATC-3’), and HPRT (fwd 5’-CTTTGCTGACCTGCTGGATTAC-3’, rev 5’-GTTGAGAGATCATCTCCACC-3’). Two pairs of ADAMTS3-specific primers were used to amplify different regions across species to compare the consistency of the qPCR results and primer pair 1 was used for final quantification. The qPCR assay was performed in triplicate.

For the CCBE1 mRNA expression analysis in MEF, NIH-3T3 and Ba/F-hVEGFR-3/EpoR cells, Taqman probes for mouse Ccbe1 and Gapdh (Applied Biosystems) were used for amplification.

Cell surface localization assay

Cos-7 cells were transfected with constructs encoding CCBE1-V5. Twenty-four hours post transfection the medium was changed to D-MEM/0.2% BSA and incubated for another 24 hours at 37°C. Cells were then washed twice with ice-cold PBS and incubated with 1M NaCl or PBS with gentle shaking for 1 hour at 4°C. Supernatants were harvested, diluted 1:10 in PBS and immunoprecipitated with anti-V5 antibody, followed by SDS-PAGE separation and detection using anti-V5 antibody.

For the analysis of VEGF-C and ADAMST3 release from the cell surface, Cos-7 cells were transfected with ADAMTS3 and VEGF-C expression constructs. After 24 hours, the media was changed to DMEM/0.2% BSA supplemented with 100 μg/ml of Heparin and incubated

for 2 or 6 hours at 37°C. The conditioned media were harvested, separated by SDS-PAGE and analyzed for VEGF-C and ADAMTS3 by Western blotting.

Detection of cell surface associated CCBE1 in lymphatic endothelial cells (LECs) was performed on confluent LECs in 60-mm dishes. LECs were washed twice with ice cold PBS and incubated with 1M NaCl or PBS for 1 hour at 4°C. Supernatants were harvested and diluted 1:10 in PBS. The dilution was used for solid-phase binding assays to detect CCBE1 using anti-CCBE1 antibody. The NaCl or PBS treated cells were washed with ice-cold PBS, lysed with reducing Laemmli buffer and analyzed by Western blotting using anti-CCBE1 antibody.

SUPPLEMENTARY FIGURES and FIGURE LEGENDS

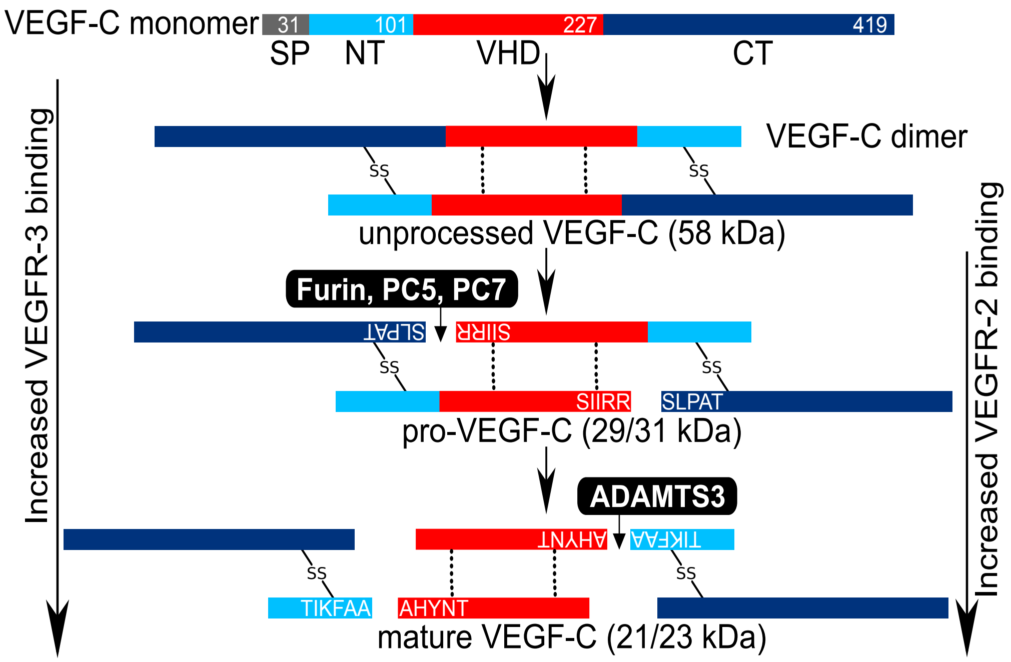

Supplementary Figure S1. Schematic view of VEGF-C synthesis and processing. The signal peptide (SP) is shown in grey, N-terminal propeptide (NT) in cyan, VEGF homology domain (VHD) in red and C-terminal (CT) propeptide in blue. The processing of VEGF-C occurs in a stepwise manner. In the first step, proprotein convertases (Furin, PC5, PC7) cleave unprocessed VEGF-C between the VHD and C-terminal propeptide. After removal of the C-terminal propeptide, the N-terminal propeptide is cleaved by ADAMTS3. The sites of amino acid cleavage are shown by arrows and adjacent amino acids in white. The mature VEGF-C has both angiogenic and lymphangiogenic activity.

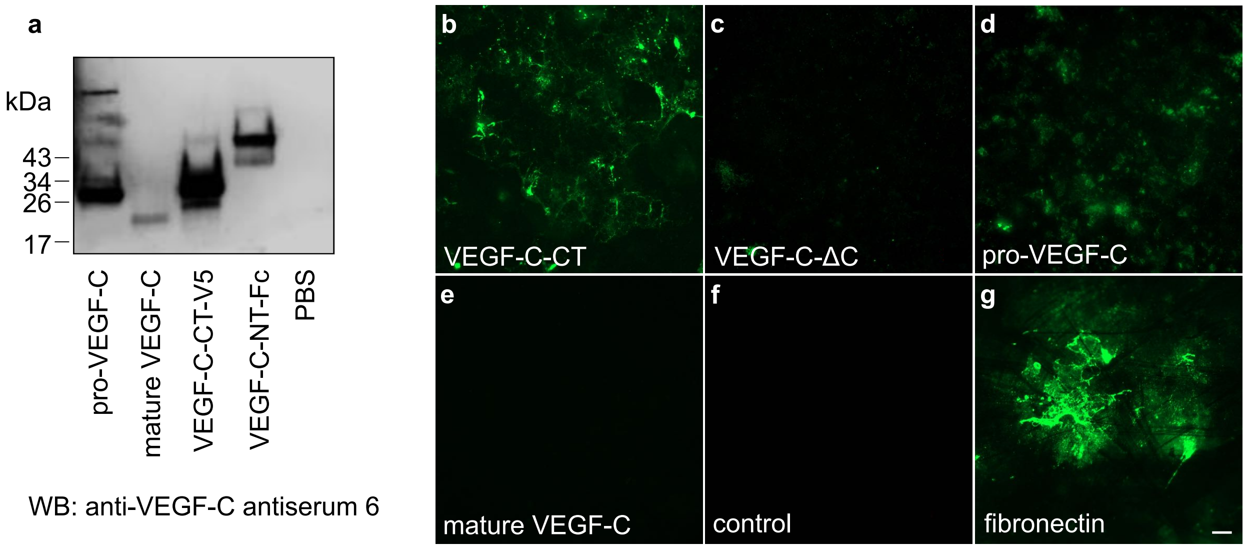

Supplementary Figure S2. pro-VEGF-C localizes to extracellular matrix (ECM). (a) Western blot of the recombinant VEGF-C forms used for matrix binding experiment with anti-VEGF-C antiserum 6. pro-VEGF-C was used at a 4-fold lower molar concentration compared to the other VEGF-C forms. (**b-g**) Immunofluorescence of cell free ECM for VEGF-C. The ECM was derived from cells expressing (**b**) VEGF-C-CT, (**c**) VEGF-C-ΔC, (**d**) pro-VEGF-C, (**e**) mature VEGF-C and (**f**) empty vector (control). (**g**) Matrix deposition was confirmed by detecting fibronectin. Scale bar, 50 μm.

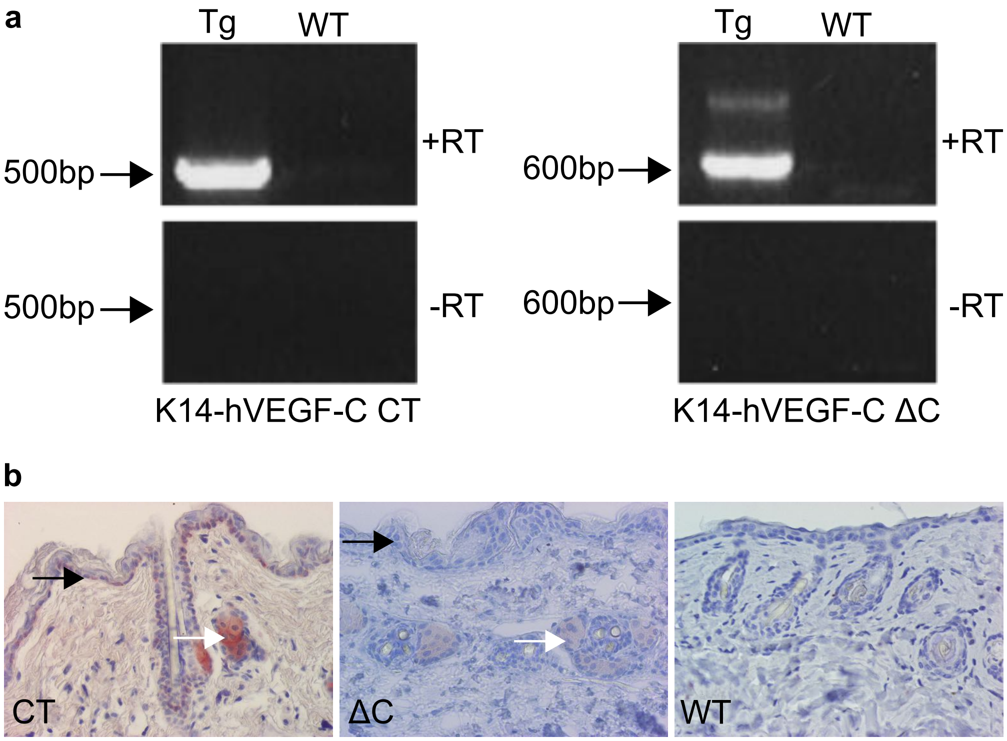

Supplementary Figure S3. Expression of VEGF-C-ΔC and VEGF-C-CT in skin of transgenic mice. (**a**) RT-PCR from embryonic skin of K14-VEGF-C-CT and K14-VEGF-C-ΔC mice from representative founder lines and of wild type littermates with (+RT) and without (-RT) reverse transcriptase. (**b**) Immunohistochemistry using antiserum against human VEGF-C (anti-VEGF-C antiserum 6) shows transgene expression in the basal layer of the epidermis (black arrows) and around hair follicles (white arrows). Note the apparent differences in the transgene expression between K14-VEGF-C-CT and K14-VEGF-C-ΔC. Because of its weak interaction with the ECM and cell surfaces, most of the VEGF-C-ΔC protein likely diffuses away during sample preparation and is therefore difficult to stain. WT mice were used as a control.

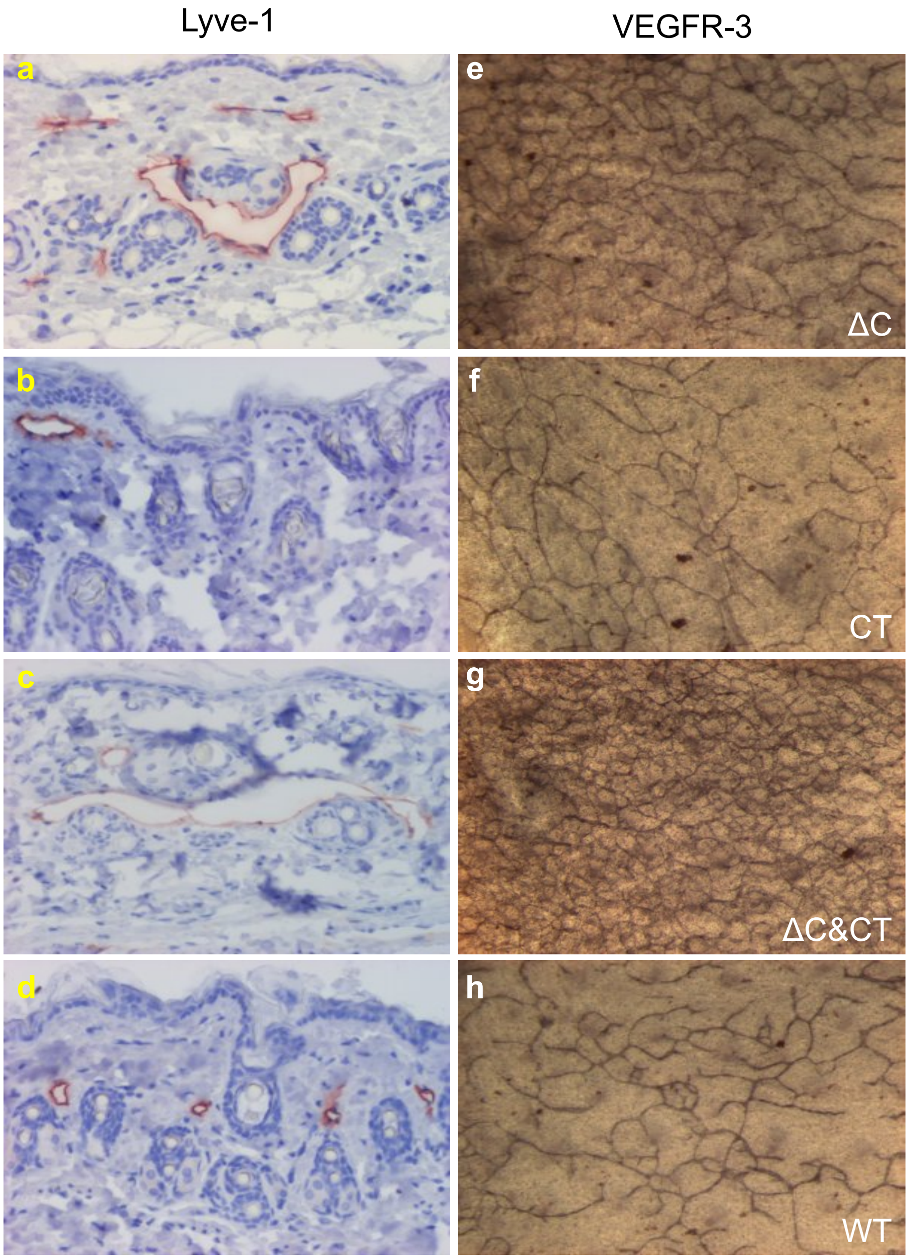

Supplementary Figure S4. VEGF-C-CT enhances lymphatic hyperplasia induced by VEGF-C-ΔC. (**a-d**) Immunohistochemical staining of skin sections for Lyve-1 and whole-mount staining of dorsal skin for VEGFR-3 of K14-VEGF-C-ΔC (**a** and **e**), K14-VEGF-CT (**b** and **f**), K14-VEGF-C-ΔC x K14-VEGF-CT double transgenic (**c** and **g**) and wild type littermates (**d** and **h**).

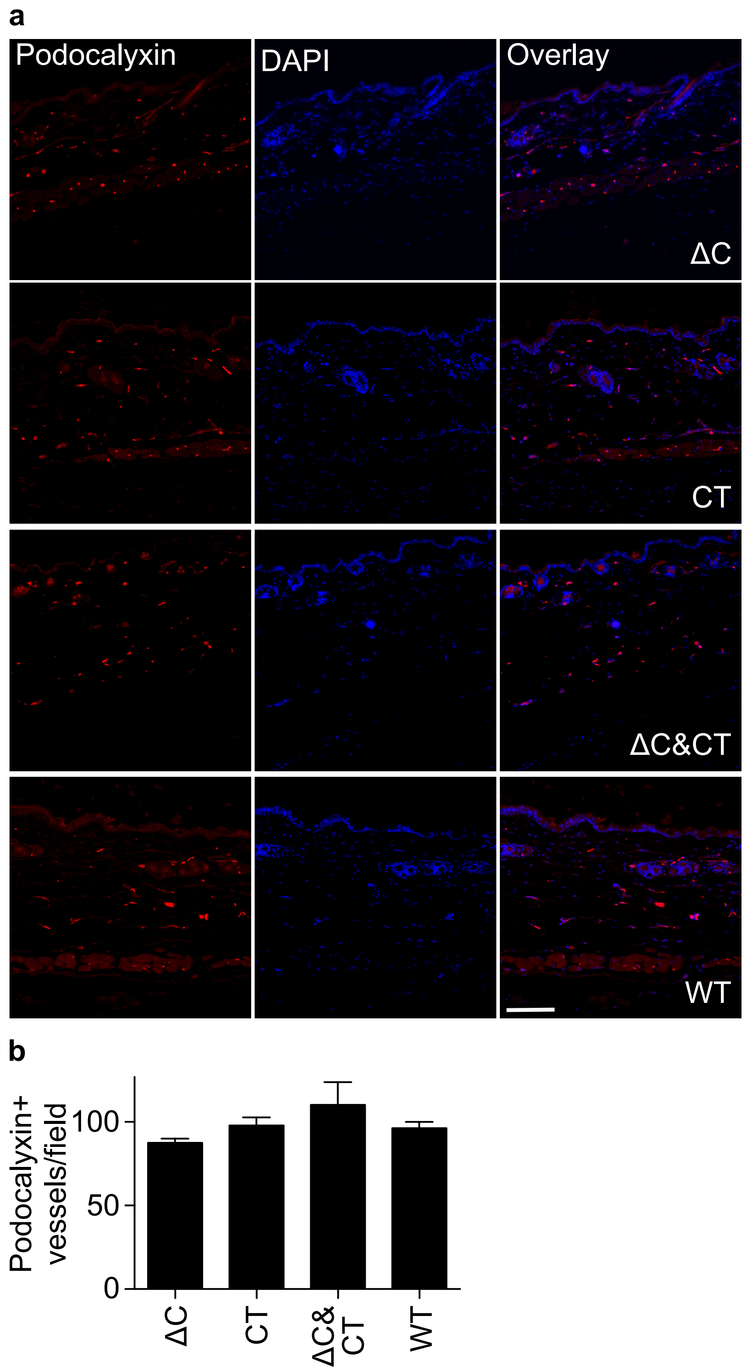

Supplementary Figure S5. Transgenic expression of VEGF-C-ΔC and VEGF-C-CT shows no effect on the blood vasculature. (**a**) Immunohistochemical staining of skin sections for podocalyxin of K14-VEGF-C-ΔC, K14-VEGF-C-CT, K14-VEGF-C-ΔC x K14-VEGF-CT double transgenic mice and wild type littermates. (**b**) Quantification of podocalyxin positive vessels per field. Differences are not significant at $p > 0.05$. Scale bar, 100 μm.

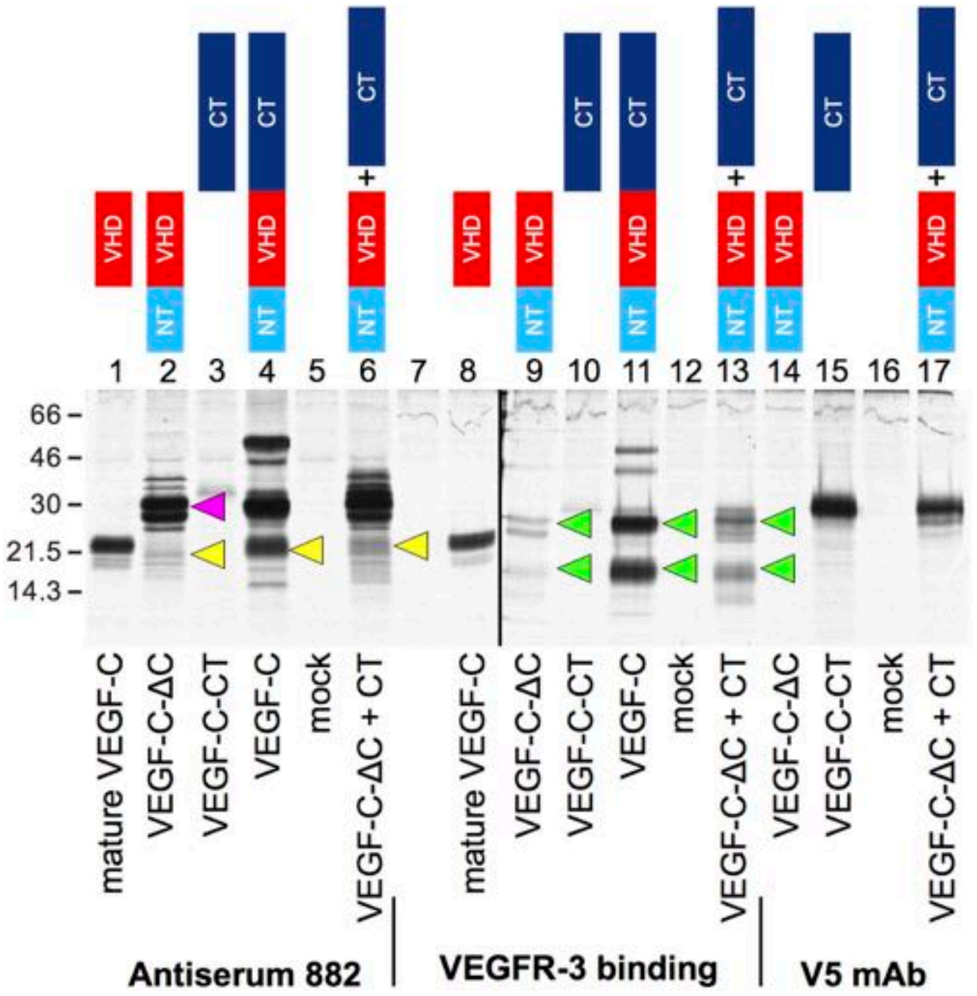

Supplementary Figure S6. The VEGF-C C-terminus rescues activation and receptor binding of VEGF-C- ΔC *in vitro*. Processing of pro-VEGF-C (magenta arrow) into mature VEGF-C (yellow arrows) and the ability to bind to VEGFR-3 (green arrows) are reduced when the C-terminus is omitted from VEGF-C. VEGF-C cleavage (lane 6) and its VEGFR-3 binding pattern (lane 13) are normalized when VEGF-C-ΔC is co-expressed with VEGF-C-CT. Metabolically labeled proteins were precipitated from the conditioned medium of transfected 293T cells with VEGFR-3-Ig fusion proteins, with antiserum 882 or with anti-V5-antibody (VEGF-C-CT is V5-tagged) and analyzed by 12% SDS-PAGE under reducing condition.

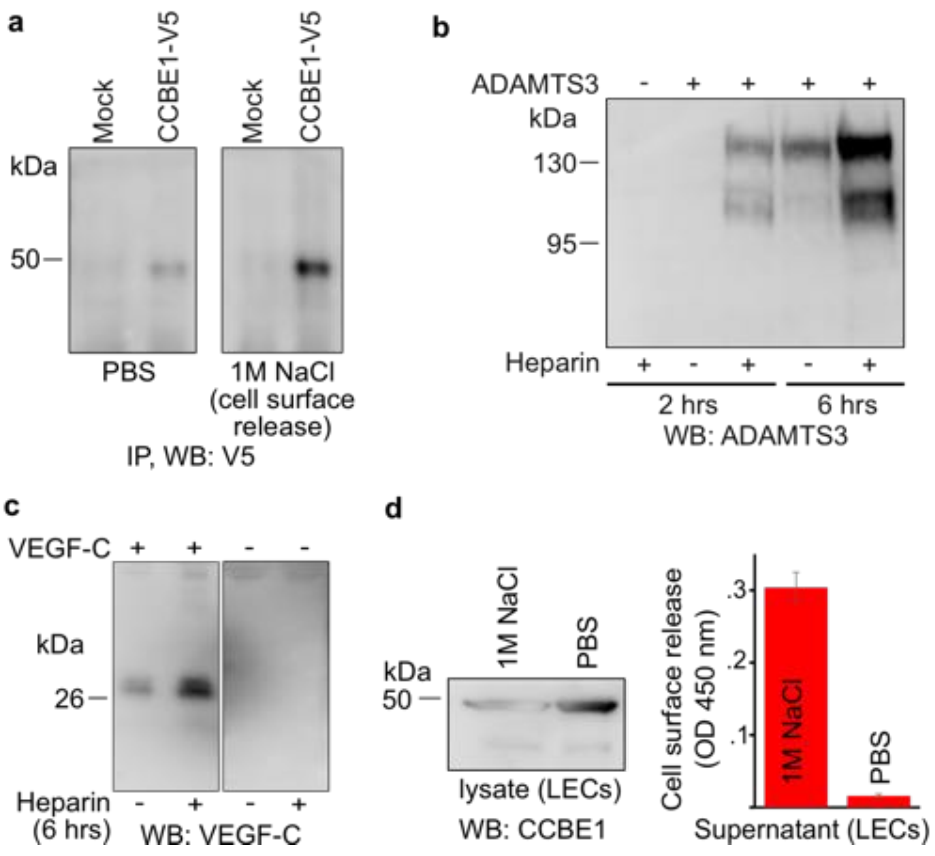

Supplementary Figure S7. CCBE1 and ADAMTS3 localize to the cell surface. (**a**) Cos-7 cells transfected with a CCBE1-V5 expression construct were treated with 1M NaCl or PBS. The released proteins were subjected to immunoprecipitation with anti-V5 antibody and analyzed by Western blotting. CCBE1 released from the cell surface by 1M NaCl lacks the chrondroitinylated (~100 kDa) form. (**b**) Cos-7 cells were transfected with an expression construct for ADAMTS3 and after 24 hours, the medium was changed to D-MEM/0.2% BSA, supplemented with 100 μg/ml heparin. 2 and 6 hours after heparin treatment, the conditioned media were harvested and analyzed for ADAMTS3 by Western blotting. (**c**) In the culture of Cos-7 cells transfected with an expression construct for VEGF-C, the ratio of cell-surface attached versus free pro-VEGF-C is a function of the heparin concentration. Conditioned cell culture supernatant was analyzed by Western blotting using anti-VEGF-C antiserum 6. (**d**) Confluent culture of lymphatic endothelial cells (LECs) were treated with 1M NaCl or PBS. The cells were lysed and analyzed by Western blotting for CCBE1. The supernatant was analyzed for its binding to anti-CCBE1 antibody coated plates in a solid-phase binding assay.

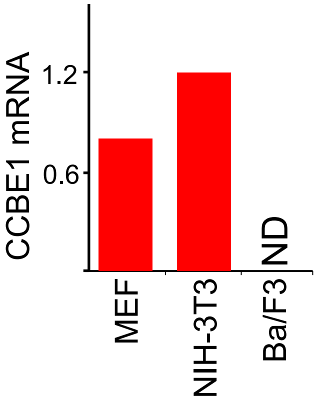

Supplementary Figure S8. Analysis of Ccbe1 mRNA levels. Expression levels were normalized to Gapdh and expressed relative to the average Ccbe1 mRNA level. ND: Not detectable.

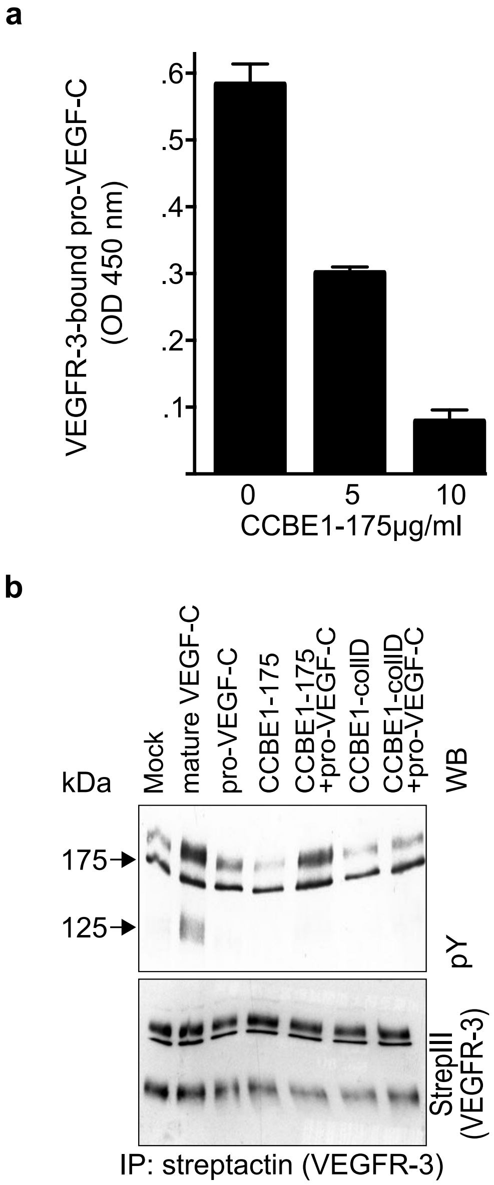

Supplementary Figure S9. The N-terminal domain of CCBE1 competes with pro-VEGF-C for VEGFR-3 binding but enhances activity of VEGFR-3 in cell based assay. (**a**) In a solid phase binding assay using purified proteins, CCBE1-175 competes with pro-VEGF-C for binding to immobilized VEGFR-3 in a concentration-dependent manner. (**b**) CCBE1-175 enhances the phosphorylation of VEGFR-3 in PAE cells exposed to pro-VEGF-C, while CCBE1-CollD has little or no effect in this assay (lanes 5 and 7). Both CCBE1-175 (lane 4) and CCBE1-CollD (lane 6) induce by themselves no or only insignificant VEGFR-3 phosphorylation compared to the control (lane 1). Shown are two Western blots from parallel runs of the same sample.

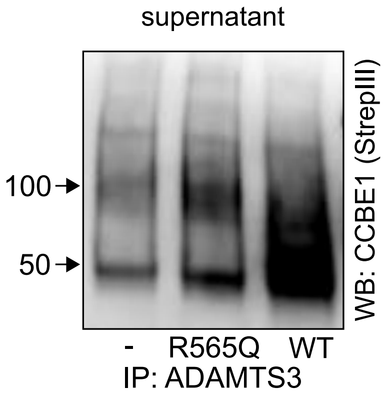

Supplementary Figure S10. The ADAMTS3 R565Q substitution affects ADAMTS3-CCBE1 interaction. Constructs coding for ADAMTS3-WT or ADAMTS3-R565Q were transfected into 293T cells stably expressing CCBE1. Lower amounts of CCBE1 were co-precipitated with ADAMTS3 from the supernatant of ADAMTS3-R565Q transfected cells compared to ADAMTS3-WT transfected cells. The small amount of co-precipitating CCBE1 in lane 1 (no ADAMTS3 transfection) likely results from endogenous ADAMTS3 expression by 293T cells and/or nonspecific binding of CCBE1 to the protein G sepharose.

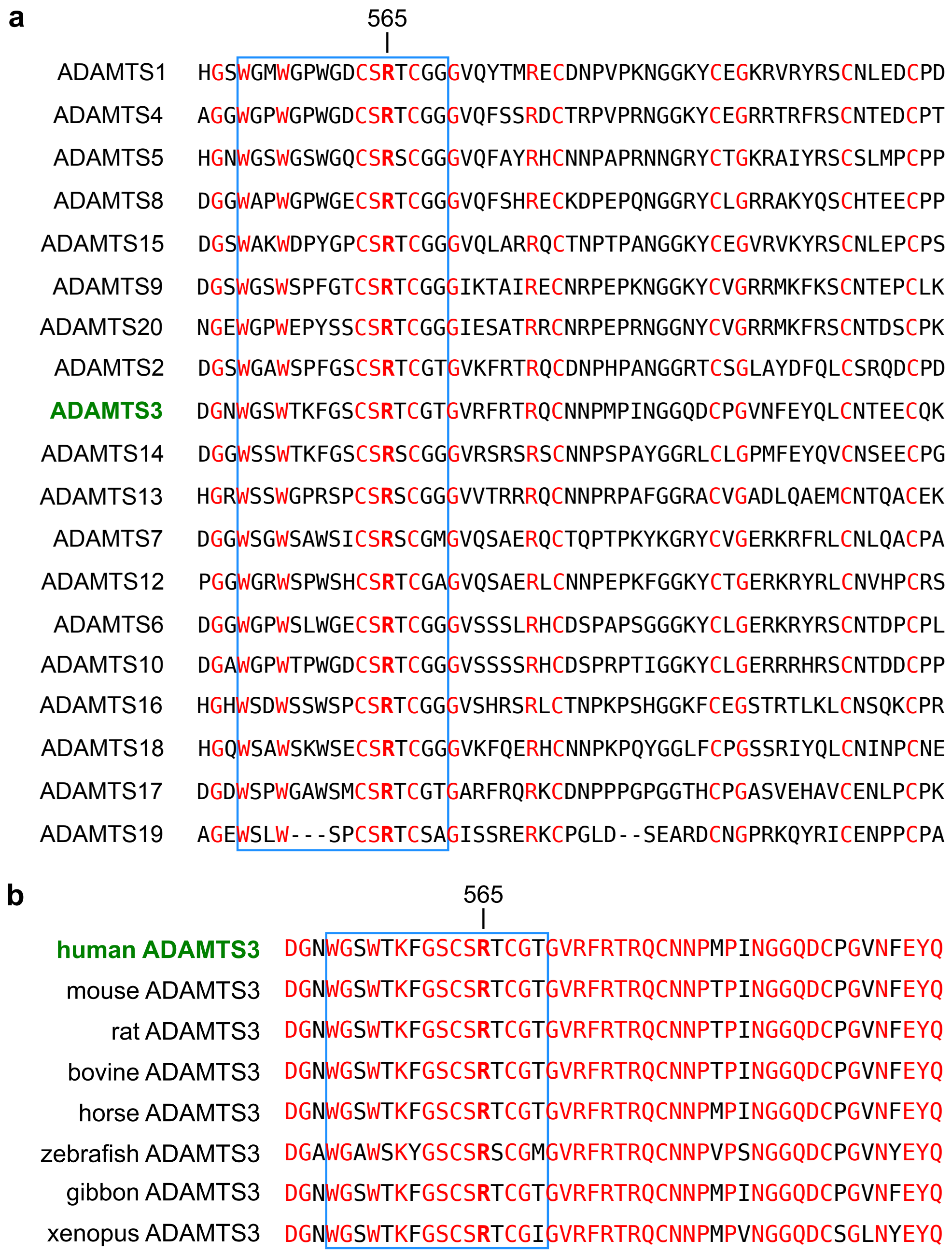

Supplementary Figure S11. Arginine 565 in ADAMTS3 is located in the highly conserved TSP-1-like region of the protein. (**a**) Alignment of the human ADAMTS3 amino acid sequence in proximity to R565 with (**a**) paralogous and (**b**) orthologous proteases. Arginine 565 is located in the highly conserved TSP-1 like domain of ADAMTS3. The motif responsible for glycosaminoglycan (GAG) and CD36 binding is boxed in blue[5]. Amino acids 100% conserved in these alignments are shown in red.

Figure 3

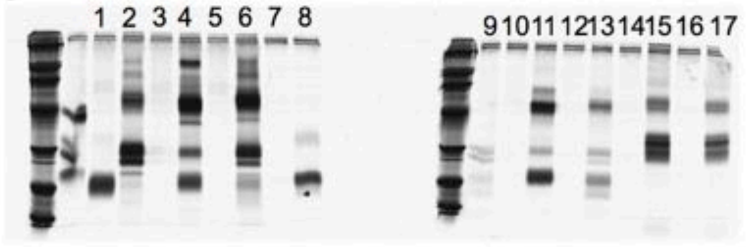

Figure 4c

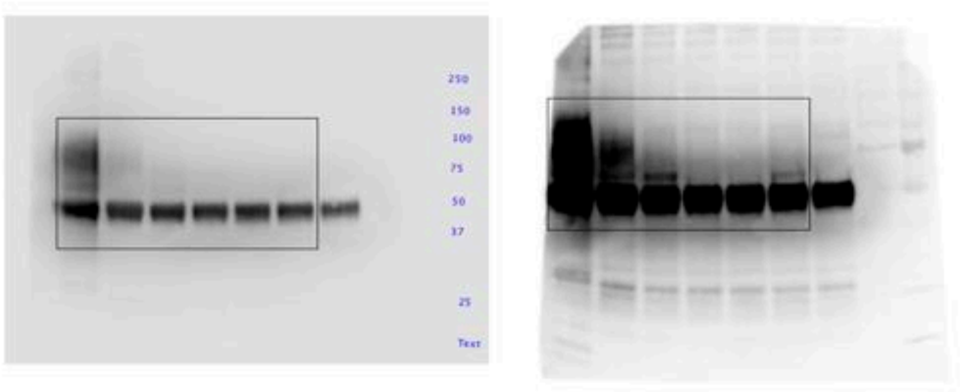

Supplementary Figure S12. Full blots for Figure 3 and 4c.

Figure 5a

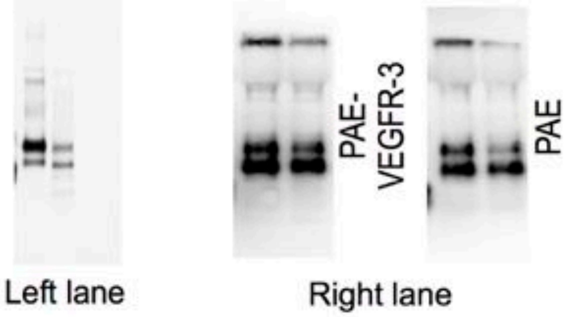

Figure 6b

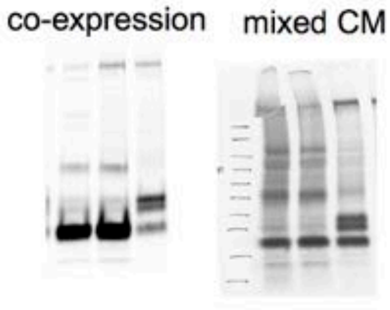

Figure 6c

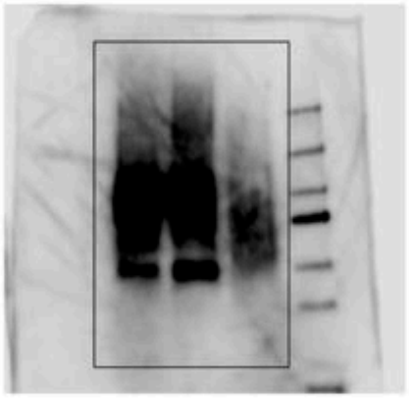

Supplementary Figure S13. Full length blots for Figure 5a, 6b and 6c.

SUPPLEMENTARY REFERENCES

1. Kärpänen, T. *et al.* Functional interaction of VEGF-C and VEGF-D with neuropilin receptors. *FASEB J. Off. Publ. Fed. Am. Soc. Exp. Biol.* **20,** 1462–1472 (2006).
2. Jeltsch, M. Hyperplasia of Lymphatic Vessels in VEGF-C Transgenic Mice. *Science* **276,** 1423–1425 (1997).
3. Jeltsch, M. *et al.* CCBE1 Enhances Lymphangiogenesis via A Disintegrin and Metalloprotease With Thrombospondin Motifs-3-Mediated Vascular Endothelial Growth Factor-C Activation. *Circulation* **129,** 1962–1971 (2014).
4. Leppanen, V.-M. *et al.* Structural and mechanistic insights into VEGF receptor 3 ligand binding and activation. *Proc. Natl. Acad. Sci.* **110,** 12960–12965 (2013).
5. Tortorella, M. *et al.* The thrombospondin motif of aggrecanase-1 (ADAMTS-4) is critical for aggrecan substrate recognition and cleavage. *J. Biol. Chem.* **275,** 25791–25797 (2000).
